# Sociodemographic inequities associated with participation in leisure-time physical activity in sub-Saharan Africa: an individual participant data meta-analysis

**DOI:** 10.1186/s12889-020-08987-w

**Published:** 2020-06-15

**Authors:** Anna Louise Barr, Uttara Partap, Elizabeth H. Young, Kokou Agoudavi, Naby Balde, Gibson B. Kagaruki, Mary T. Mayige, Benjamin Longo-Mbenza, Gerald Mutungi, Omar Mwalim, Chea S. Wesseh, Silver K. Bahendeka, David Guwatudde, Jutta M. Adelin Jørgensen, Pascal Bovet, Ayesha A. Motala, Manjinder S. Sandhu

**Affiliations:** 1 Department of Medicine, University of Cambridge, Cambridge, UK; 2 Wellcome Sanger Institute, Genome Campus, Hinxton, UK; 3 Togo Ministry of Health, Lome, Togo; 4 Department of Endocrinology and Diabetes, Donka University Hospital, Conakry, Guinea; 5 National Institute for Medical Research, Tukuyu Research Centre, Tukuyu, Tanzania; 6 National Institute for Medical Research, Headquarter Research Centre, Dar es Salaam, Tanzania; 7 Faculty of Health Sciences, Walter Sisulu University, Mthatha, Eastern Cape South Africa; 8 LOMO University of Research, Kinshasa, Democratic Republic of Congo; 9 Control of Non-Communicable Diseases Desk, Ministry of Health, Kampala, Uganda; 10 Zanzibar Ministry of Health, Mnazi Mmoja, Tanzania; 11 Ministry of Health, Monrovia, Liberia; 12 Mother Kevin Postgraduate Medical School (MKPGMS), Uganda Martyrs University, Kampala, Uganda; 13 St. Francis Hospital, Nsambya, Kampala, Uganda; 14 Department of Epidemiology and Biostatistics, School of Public Health, Makerere University, Kampala, Uganda; 15 Department of Public Health, University of Copenhagen, Copenhagen, Denmark; 16 University Center for Primary Care and Public Health (Unisanté), Lausanne, Switzerland; 17 Ministry of Health, Victoria, Republic of Seychelles; 18 Department of Diabetes and Endocrinology, Nelson R. Mandela School of Medicine, University of KwaZulu-Natal, Durban, South Africa

**Keywords:** Leisure-time physical activity, Physical activity, Sub-Saharan Africa, Occupational physical activity, Active travel, Global physical activity questionnaire, Recreation, Equity, Urbanisation, Mechanisation

## Abstract

**Background:**

Leisure-time physical activity (LTPA) is an important contributor to total physical activity and the focus of many interventions promoting activity in high-income populations. Little is known about LTPA in sub-Saharan Africa (SSA), and with expected declines in physical activity due to rapid urbanisation and lifestyle changes we aimed to assess the sociodemographic differences in the prevalence of LTPA in the adult populations of this region to identify potential barriers for equitable participation.

**Methods:**

A two-step individual participant data meta-analysis was conducted using data collected in SSA through 10 population health surveys that included the Global Physical Activity Questionnaire. For each sociodemographic characteristic, the pooled adjusted prevalence and risk ratios (RRs) for participation in LTPA were calculated using the random effects method. Between-study heterogeneity was explored through meta-regression analyses and tests for interaction.

**Results:**

Across the 10 populations (*N* = 26,022), 18.9% (95%CI: 14.3, 24.1; *I*^2^ = 99.0%) of adults (≥ 18 years) participated in LTPA. Men were more likely to participate in LTPA compared with women (RR for women: 0.43; 95%CI: 0.32, 0.60; *P* < 0.001; *I*^2^ = 97.5%), while age was inversely associated with participation. Higher levels of education were associated with increased LTPA participation (RR: 1.30; 95%CI: 1.09, 1.55; *P* = 0.004; *I*^2^ = 98.1%), with those living in rural areas or self-employed less likely to participate in LTPA. These associations remained after adjusting for time spent physically active at work or through active travel.

**Conclusions:**

In these populations, participation in LTPA was low, and strongly associated with sex, age, education, self-employment and urban residence. Identifying the potential barriers that reduce participation in these groups is necessary to enable equitable access to the health and social benefits associated with LTPA.

## Background

Physical activity can be undertaken within several domains of an individual’s life, with work, travel and leisure-time the most commonly defined. In many high- and middle-income countries, increasing mechanisation and the rise in sedentary employment have led to notable declines in the time spent in occupational physical activity [1, 2]. Likewise, urbanisation and increasing dependence on motor vehicles has led to a reduction in the amount of physical activity accumulated through active travel [3].

Conversely, many of these same countries have observed an increase in the contribution of leisure-time physical activity (LTPA) to total physical activity [1]. Globally, LTPA and its associated market is worth billions, [4] and is an important target for public health interventions attempting to reduce the epidemic of insufficient physical activity amongst populations [5]. Importantly, a number of prospective studies and meta-analyses have indicated that LTPA may have greater health benefits compared with other domains of physical activity [6–8]. However, not all groups are engaged in regular LTPA: women, older adults, and low-income or less educated populations are often less likely to participate [9, 10]. Many high-income countries (HICs) are working hard to identify and tackle the social, economic and environmental barriers which prevent these groups from participating in regular LTPA, and to ensure equitable access to the associated health and social benefits [11–13].

By contrast, knowledge of the physical activity patterns of populations in sub-Saharan Africa (SSA) is limited. Africa is the fastest urbanising continent, [14] and in recent decades has experienced rapid lifestyle changes, mirroring those observed in higher income countries. A recent review illustrated that for sub-Saharan African countries with lower levels of physical activity, the contribution of occupational physical activity to total physical activity was reduced, while the contribution of active travel increased [15]. The contribution of LTPA was low across all countries [15]. However, this review did not estimate the prevalence of participation across the different domains of physical or assess the differences in participation across population subgroups.

With increasing economic development, the contribution of LTPA to total physical activity is likely to rise in sub-Saharan African populations; however, currently little is known about those participating in LTPA. Using data from 10 diverse populations across SSA, we sought to address the current gap in understanding by assessing the prevalence of participation in LTPA, and the associated sociodemographic factors, to help identify the groups at risk of exclusion and potential barriers to participation within the African context.

## Methods

### Survey methods

As part of the ongoing collaborative work of the African Partnership for Chronic Disease Research, individual participant data (IPD) from 10 large-scale adult population surveys were collated for the purpose of these analyses. These surveys were conducted in nine countries: Democratic Republic of Congo (DRC), [16] Guinea, [17] Kenya, [18] Liberia, [19] Seychelles, [20] South Africa, [21] United Republic of Tanzania (consisting of Tanzania and Zanzibar), [22, 23] Togo, [24] and Uganda [25]. All studies utilised or were consistent with the WHO STEPwise approach to non-communicable disease risk factor surveillance tool (STEPS), which includes the Global Physical Activity Questionnaire (GPAQ) [26]. The methods of each survey are described in greater detail elsewhere [16, 17, 19–25, 27]. Each study employed a multi-level sampling strategy (Supplementary Table S1), and was designed to be representative of the national or subnational populations from which they were drawn. Surveys were conducted through face-to-face interviews, with all surveys apart from Liberia having responses greater than 70.0% (Supplementary Table S1).

### Definition of exposure and outcomes

Age, in years, was recoded into six categories: 18–24, 25–34, 35–44, 45–54, 55–64, and 65 and above. Education was grouped into four categories based on the highest education level completed: primary education not completed, primary education completed, secondary education completed, and tertiary education completed (Supplementary methods). Employment status was re-categorised from nine to four categories (Supplementary methods): public or private employees; self-employed; non-income workers; and no occupation. Participants’ height and weight measurements were used to calculate their body-mass index (BMI). BMI was categorised into four categories based on standard cut-offs: underweight (BMI: < 18.5 kg/m^2^); healthy weight (BMI: 18.5–24.9 kg/m^2^); overweight (BMI: 25.0–29.9 kg/m^2^); and obese (BMI: ≥ 30.0 kg/m^2^). Six studies collected data on urban or rural residence.

Physical activity variables were cleaned according to the GPAQ guidelines [28]. Insufficient physical activity was defined as undertaking less than 600 metabolic equivalent (MET) minutes of physical activity per week, [29] while participation in physical activity was defined as undertaking > 0 MET-minutes of physical activity per week. Participation in LTPA, occupational physical activity or active travel was defined as undertaking > 0 MET-minutes of physical activity within these domains.

### Statistical analyses

We conducted a two-step IPD meta-analysis, analysing each dataset separately to obtain study-level estimates, before combining them using random effects models of meta-analysis. Participants were included if individuals were aged 18 years and above and had complete information on sex, age, education, employment, BMI, physical activity and sampling units. All analyses were conducted using Stata/SE 14.2 (StataCorp, Texas).

Adjusted prevalence estimates of the sociodemographic characteristics investigated were calculated for each individual study population using mixed effects Poisson regression models with robust standard errors, adjusted for sex, age and clustering at each level of sampling (Supplementary Table S2). Adjusted prevalence estimates for participation in physical activity within each domain overall, and for each sociodemographic group, were then calculated for each individual study using mixed effects Poisson regression models with robust standard errors, adjusted for sex, age and clustering at each level of sampling. Using the *metaprop* Stata command, which is specifically designed to pool proportions, pooled adjusted prevalence estimates for sociodemographic characteristics and domain participation were calculated using a random effects model [30]. The *metaprop* command requires a total for each sub-group (N) and a number of the participants within that sub-group with the condition (n). The predicted n was calculated by multiplying the individual country adjusted prevalence estimates against the N for that study–the total number of participants in each study with complete data. The study-specific confidence intervals were calculated using the exact method [30]. The outcomes were normalised using the Freeman-Tukey double arcsine transformation and the pooled adjusted prevalence estimates were computed using the DerSimonian and Laird method [31]. The confidence intervals of the pooled adjusted prevalence estimates were calculated using the Wald method [30].

Poisson regression models, with robust standard errors, were fitted to obtain study-specific risk ratios (RRs) for participation in each domain of physical activity. Mixed effects accounted for the clustered nature of the data. Fully-adjusted models were adjusted for sex, age, education, employment and BMI. The pooled RRs were calculated for each sociodemographic characteristic using a weighted average of the log-adjusted RR, allowing for random effects using the DerSimonian and Laird method [31].

Sensitivity analyses were conducted to check whether a single study substantially influenced the pooled RRs, by excluding each study from the pooled analyses in turn and comparing results with and without the study in question. In supplementary analyses, we investigated whether adjustment for participation in physical activity at work or through travel, or the volume of physical activity accumulated in each domain, affected observed associations with participation in LTPA.

The I^2^ statistic was used to assess the heterogeneity between study-specific estimates e.g. pooled adjusted prevalence estimates and RRs [32]. We considered values of ≥75% to indicate high heterogeneity, values < 75 and > 25% to indicate moderate heterogeneity and values of ≤25% to indicate low heterogeneity [33]. Effect modification was assessed by stratifying the fully-adjusted model by sex, education and residence location (urban/rural). We also assessed whether there was statistical evidence of interaction between sex and all other covariates, education and all other covariates, and residence location and all other covariates, regarding their association with participation in LTPA. This was done by including an interaction term within the fully-adjusted model and pooling the log RR for the interaction term using the method of DerSimonian and Laird described above.

Finally, random effects meta-regression was used to explore the influence of study design and population characteristics, listed in Table 1, on the between-study heterogeneity for the fully-adjusted models. Additionally, as the calculated *P*-values using this method tend to be conservative, we conducted permutation tests based on Monte Carlo simulation, adjusting the *P*-values for multiple-testing [34].

**Table 1:** Study design and population characteristics of included population health surveys

**Study (reference)**	**Study period**	**Geographical scope**	**Sample size**^**a**^ **(N)**	**Income group**	**HDI**	**GDP per capita (US$)**	**Urban population (%)**
DRC [13]	2005	Subnational	1502	Low	0.364	218.5	37.5
Guinea [14]	2009	Subnational	2125	Low	0.380	615.1	34.4
Togo [21]	2010	National	2051	Low	0.457	487.9	37.5
Liberia [16]	2011	National	2206	Low	0.416	379.7	48.2
Zanzibar [20]	2011	Subnational	2640	Low	0.504	733.4	28.8
Tanzania [19]	2012	National	5525	Low	0.513	820.2	29.5
Seychelles [17]	2013	National	1232	Upper-middle	0.766	14,764.9	53.2
South Africa [18]	2013	Subnational	1014	Upper-middle	0.660	6822.5	63.8
Uganda [22]	2014	National	3543	Low	0.488	702.8	15.8
Kenya [15]	2015	National	4184	Low-middle	0.555	1355.0	25.6

*DRC* Democratic Republic of Congo, *Income group* World Bank income group, *HDI* Human Development Index, *GDP per capita* Global domestic product per capita, *Urban population*: Percentage of the total population living in urban areas.

World Bank income group, HDI, GDP per capita and urban population data relates to year of study [30–33], Zanzibar taken from data reported for Tanzania in 2011 as no regional specific data was available.

^a^Sample size is the sample size after data cleaning was completed and those with no or invalid information on variables of interest removed

### Ethics

Each primary study obtained ethical approvals in its respective country along with informed consent from participants. This study received ethical approval from the Human Biology Research Ethics Committee at the University of Cambridge, UK (Application No: HBREC.2015.05).

## Results

The studies included in this IPD meta-analysis were undertaken over a period of 10 years and were a mixture of representative surveys from national and subnational populations (*N* = 26,022) (Table 1). The studies were conducted in countries at various stages of economic and urban development [35–38].

Women made up 59.5% (95% confidence interval (CI): 56.7, 62.3; *I*^*2*^ = 95.3%) of the population (Table 2). The majority of individuals were aged between 25 and 44 years. A small proportion of the population was educated to tertiary level and 38.5% (95%CI: 27.5, 50.0; *I*^*2*^ = 99.7%) were self-employed. Over one third of the population were classified as overweight or obese. For those studies which reported residence location, the majority of individuals lived in rural areas (60.8%; 95%CI: 48.3, 72.6; *I*^*2*^ = 99.7%). The pooled prevalence of insufficient physical activity was 9.5% (95%CI: 6.3, 13.4; *I*^*2*^ = 99.0%); however at least 94.1% (95%CI: 91.1, 96.5; *I*^*2*^ = 98.8%) of the population undertook some form of physical activity per week. Overall, the prevalence of participation in LTPA was 18.9% (95%CI: 14.3, 24.1; *I*^*2*^ = 99.0%) (Table 2). LTPA participation ranged from 1.6% (95%CI: 0.7, 2.4) in DRC to 27.1% (95%CI: 24.7, 29.4) in Tanzania (Supplementary Table S3). By contrast, the pooled adjusted prevalence of participation in physical activity within the domains of work and travel were 70.1% (95%CI: 53.5, 84.4; *I*^*2*^ = 99.9%) and 86.2% (95%CI: 82.5, 89.5; *I*^*2*^ = 98.5%), respectively (Table 2 and Supplementary Figures S1-S2).

**Table 2:** Pooled adjusted prevalence of sociodemographic characteristics (*N* = 26,022)

**Characteristic**	N	**% (95%CI)**	***I***^***2***^
**Sex**^**a**^			
Men	10	40.1 (37.3, 42.9)	95.2%
Women	10	59.5 (56.7, 62.3)	95.3%
**Age group**^**b**^**(years)**			
18–24	9	13.0 (5.3, 23.4)	99.8%
25–34	10	29.9 (27.2, 32.5)	95.4%
35–44	10	22.4 (19.6, 25.3)	96.7%
45–54	10	17.0 (14.2, 20.0)	97.5%
55–64	10	12.1 (9.5, 15.1)	97.9%
65+	6	2.6 (0.6, 5.9)	99.2%
**Education**			
Primary education not completed	9	30.2 (19.2, 42.4)	99.8%
Primary education completed	10	25.7 (15.3, 37.9)	99.8%
Secondary education completed	10	30.4 (17.8, 44.6)	99.8%
Tertiary education completed	10	4.0 (2.4, 5.9)	98.2%
**Employment**			
Private or public employee	10	16.0 (9.4, 24.0)	99.6%
Self-employed	10	38.5 (27.5, 50.0)	99.7%
Non-income work	10	23.6 (16.9, 31.0)	99.5%
No occupation	10	9.4 (4.6, 15.7)	99.6%
**BMI (kg/m**^**2**^**)**			
Underweight	10	7.8 (5.6, 10.3)	97.9%
Healthy weight	10	53.7 (47.2, 60.2)	99.1%
Overweight	10	21.0 (17.7, 24.4)	97.7%
Obese	10	12.5 (8.0, 17.8)	99.3%
**Residence location†**^**c**^			
Urban	6	39.2 (27.4, 51.7)	99.7%
Rural	6	60.8 (48.3, 72.6)	99.7%
**Physical activity (MET-mins/wk)**			
Insufficient	10	9.5 (6.3, 13.4)	99.0%
**Participation in physical activity (MET-mins/wk)**			
Participation in physical activity (all domains)	10	94.1 (91.1, 96.5)	98.8%
Leisure-time physical activity (LTPA)	10	18.9 (14.3, 24.1)	99.0%
Occupational physical activity	10	70.1 (53.5, 84.4)	99.9%
Active travel	10	86.2 (82.5, 89.5)	98.5%

N: number of studies; %: Pooled adjusted prevalence; 95%CI: 95% confidence interval; *I*^*2*^: *I*^*2*^ statistic indicates proportion of variation due to heterogeneity; MET-mins/wk: Metabolic Equivalent minutes per week; Insufficient: < 600 MET-minutes per week; Participation in physical activity: > 0 MET-minutes of physical activity per week undertaken in all domains; Leisure-time physical activity: > 0 MET-minutes spent in leisure-time physical activity per week; Occupational physical activity: > 0 MET-minutes spent in occupational physical activity per week; Active travel: > 0 MET-minutes spent in physical activity through active travel per week; Underweight (BMI: < 18.5 kg/m^2^); Healthy weight (BMI: 18.5–24.9 kg/m^2^); Overweight (BMI: 25.0–29.9 kg/m^2^); Obese (BMI: ≥ 30.0 kg/m^2^)

^†^Residence location: *N* = 20,022

Adjusted prevalence estimates are based on Poisson regression models adjusted for sex, age and clustering at each level of sampling

^a^Adjusted prevalence estimates for sex are based on Poisson regression models adjusted for age and clustering at each level of sampling

^b^Adjusted prevalence estimates for age group are based on Poisson regression models adjusted for sex and clustering at each level of sampling

^c^Adjusted prevalence estimates for age group are based on Poisson regression models adjusted for sex and age

Prevalence estimates may not add up to 100% due to the effects of adjustment

**Fig. 1:**
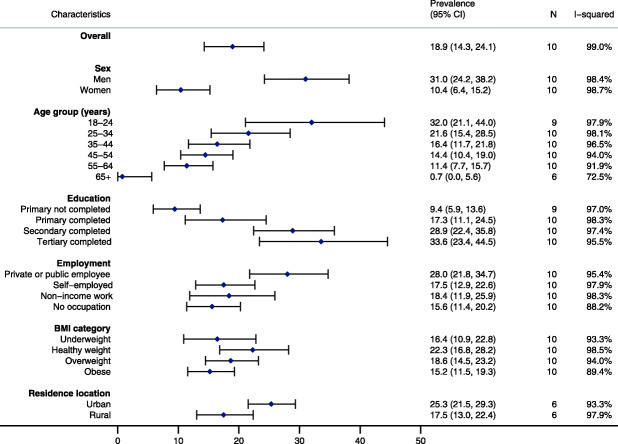
Pooled adjusted prevalence of participation in leisure-time physical activity (N = 26,022). Prevalence: pooled adjusted prevalence; 95%CI: 95% confidence interval; N: number of studies; I-squared: *I*^*2*^ statistic indicates proportion of variation due to heterogeneity; Underweight (BMI: < 18.5 kg/m^2^); Healthy weight (BMI: 18.5–24.9 kg/m^2^); Overweight (BMI: 25.0–29.9 kg/m^2^); Obese (BMI: ≥ 30.0 kg/m^2^). Residence location: N = 20,022. Pooled adjusted prevalence estimates are based on two-step individual pooled data meta-analysis of Poisson regression models adjusted for sex, age and clustering at each level of sampling. Sex: pooled adjusted prevalence estimates for sex are based on two-step individual pooled data meta-analysis of Poisson regression models adjusted for age and clustering at each level of sampling. Age group: pooled adjusted prevalence estimates for age group are based on two-step individual pooled data meta-analysis of Poisson regression models adjusted for sex and clustering at each level of sampling.

A notably higher proportion of men participated in LTPA compared with women, with participation in LTPA decreasing substantially with age (Fig. 1). We also observed that the proportion of the population participating in LTPA increased with education level. A greater proportion of urban residents reported participating in LTPA compared with rural residents. By contrast, prevalence estimates were generally similar across sociodemographic groups for participation in occupational physical activity or active travel (Supplementary Figures S1-S2 and Supplementary Table S4-S5).

**Fig. 2:**
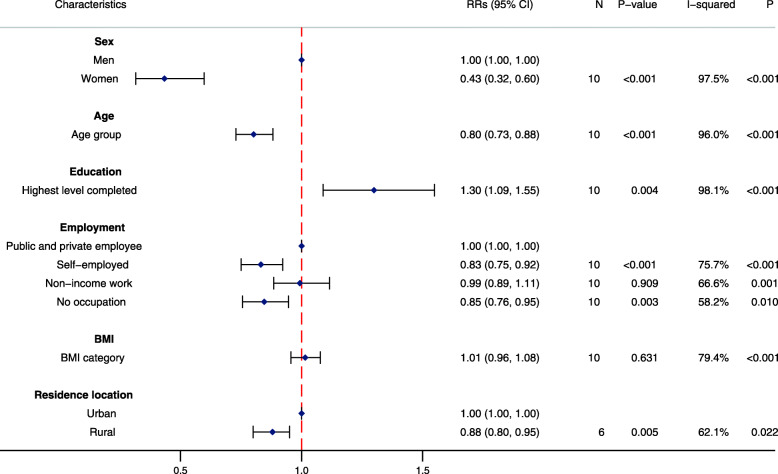
Association between sociodemographic characteristics and participation in leisure-time physical activity (*N* = 26,022). RR: Pooled risk ratio; 95%CI: 95% confidence interval; N: number of studies in two-step individual participant data meta-analysis; *P*-value: corresponds to the Z-test of significance for the pooled RR; I-squared: *I*^*2*^ statistic indicates proportion of variation due to between-study heterogeneity; P: corresponds to the *P*-value for the chi-squared test of heterogeneity. Pooled RRs calculated from two-step individual participant data meta-analysis of individual study RRs, estimated by multivariable mixed effects Poisson regression models with robust standard errors, adjusted for levels of clustering and all other covariates in the figure except residence location. Categorical variables for age, education and BMI were included in the model as continuous variables to reduce the degrees of freedom and enable model convergence. Residence location: *N* = 20,022. Men were the reference for women; public and private employees were the reference for employment; urban residents were the reference for rural residents

In fully-adjusted Poisson regression analyses, men were more likely to participate in LTPA compared with women (RR for women: 0.43, 95%CI: 0.32, 0.60; *P* < 0.001; *I*^*2*^ = 97.5%; *P* for heterogeneity < 0.001) (Fig. 2). Urban residents were more likely to participate in LTPA compared with rural residents (RR for rural: 0.88; 95%CI: 0.80, 0.95; *P* = 0.005; *I*^2^ = 62.1%; *P* for heterogeneity = 0.022). A strong positive relationship between education and participation in LTPA was observed, while a strong inverse relationship between age and participation in LTPA was also detected. Being self-employed or having no occupation was associated with a notably reduced likelihood of participating in LTPA when compared with those in public or private employment.

**Fig. 3:**
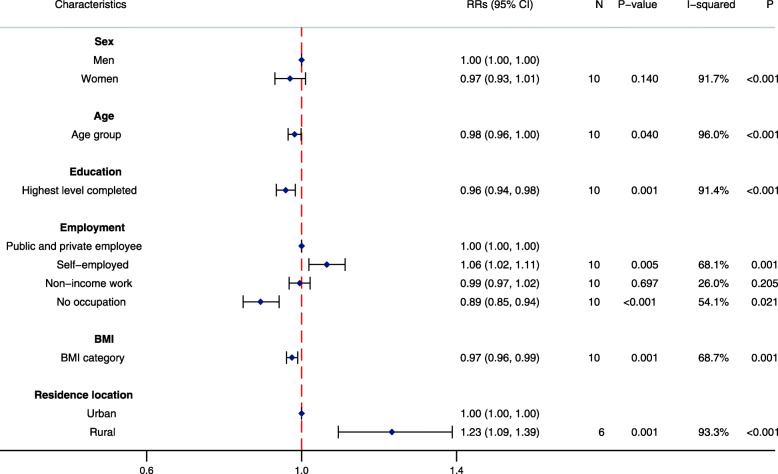
Association between sociodemographic characteristics and participation in occupational physical activity (*N* = 26,022). RR: Pooled risk ratio; 95%CI: 95% confidence interval; N: number of studies in two-step individual participant data meta-analysis; *P*-value: corresponds to the Z-test of significance for the pooled RR; I-squared: *I*^*2*^ statistic indicates proportion of variation due to between-study heterogeneity; P: corresponds to the *P*-value for the chi-squared test of heterogeneity. Pooled RRs calculated from two-step individual participant data meta-analysis of individual study RRs, estimated by multivariable mixed effects Poisson regression models with robust standard errors, adjusted for levels of clustering and all other covariates in the figure except residence location. Categorical variables for age, education and BMI were included in the model as continuous variables to reduce the degrees of freedom and enable model convergence. Residence location: *N* = 20,022. Men were the reference for women; public and private employees were the reference for employment; urban residents were the reference for rural residents

**Fig. 4:**
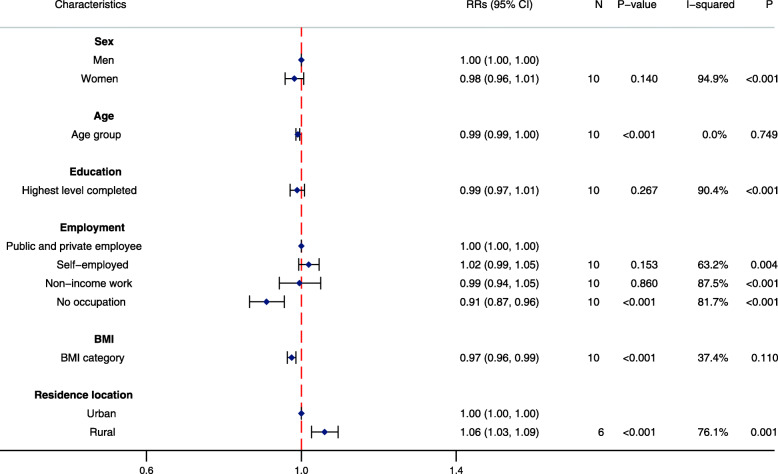
Association between sociodemographic characteristics and participation in active travel (*N* = 26,022). RR: Pooled risk ratio; 95%CI: 95% confidence interval; N: number of studies in two-step individual participant data meta-analysis; P-value: corresponds to the Z-test of significance for the pooled RR; I-squared: *I*^*2*^ statistic indicates proportion of variation due to between-study heterogeneity; P: corresponds to the *P*-value for the chi-squared test of heterogeneity. Pooled RRs calculated from two-step individual participant data meta-analysis of individual study RRs, estimated by multivariable mixed effects Poisson regression models with robust standard errors, adjusted for levels of clustering and all other covariates in the figure except residence location. Categorical variables for age, education and BMI were included in the model as continuous variables to reduce the degrees of freedom and enable model convergence. Residence location: *N* = 20,022. Men were the reference for women; public and private employees were the reference for employment; urban residents were the reference for rural residents

By contrast, few sociodemographic characteristics were strongly associated with participation in physical activity at work and through active travel in these populations (Figs. 3 and 4). Education was inversely associated with participation in occupational physical activity. Individuals who were self-employed had a slightly increased likelihood of participating in occupational physical activity when compared with public and private employees, while those with no occupation had a slightly reduced likelihood in participating in physical activity at work or through travel. Unlike LTPA, BMI was inversely associated with reduced participation in physical activity at work and through active travel. Notably, rural residents were more likely to participate in work and travel physical activity compared with urban residents.

Sensitivity analyses revealed no evidence of outlier studies and the interpretation of the RRs for participation in work, travel and leisure-time physical activity remained unchanged regardless of which study was excluded from the pooled analyses (Supplementary Tables S6, S7, S8). Adjusting for participation in occupational physical activity or active travel, or both, did not notably change the RRs for participation in LTPA (Supplementary Figures S3, S4, S5, S6, S7, S8). Likewise, the RRs remained unchanged when total MET-minutes spent in occupational physical activity or active travel, or both, were included in the model (Supplementary Figures S3, S4, S5, S6, S7, S8).

Due to low statistical power (*n* = 10), we were unable to detect substantive evidence that any of the study level or population characteristics listed in Table 1 explained the high levels of between-study heterogeneity observed in these analyses (Supplementary Tables S10, S11, S12).

**Fig. 5:**
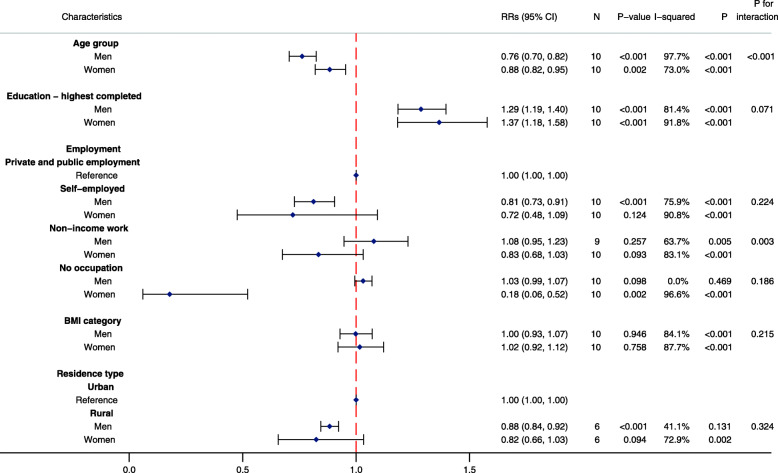
Association between sociodemographic characteristics and participation in leisure-time physical activity by sex. Men: *N* = 10,878; Women: *N* = 15,144. RR: Pooled risk ratio; 95%CI: 95% confidence interval; N: number of studies in two-step individual participant data meta-analysis; *P*-value: corresponds to the Z-test of significance for the pooled RR; I-squared: *I*^*2*^ statistic indicates proportion of variation due to between-study heterogeneity; P: corresponds to the *P* value for the chi-squared test of heterogeneity; P for interaction: corresponds to the Z-test of significance for the pooled interaction term. Pooled RRs calculated from two-step individual participant data meta-analysis of individual study RRs, estimated by multivariable mixed effects Poisson regression models with robust standard errors, adjusted for levels of clustering and all other covariates in the figure except residence location. Categorical variables for age, education and BMI were included in the model as continuous variables to reduce the degrees of freedom and enable model convergence. Residence location: *N* = 20,022. Public and private employees were the reference for employment; urban residents were the reference for rural residents

To further explore the association between sociodemographic characteristics and participation in LTPA, we stratified our models by sex, education and residence location. We observed evidence of a strong interaction between age and sex in the association with LTPA participation (*P* for interaction < 0.001) (Fig. 5). Additionally, rural men were less likely to participate in LTPA compared with urban men; however, no notable differences were observed between urban and rural women. Sensitivity analyses revealed that the RRs for women with no occupation in Guinea strongly influenced the pooled RR, with no notable difference between the sexes observed when Guinea was excluded from the pooled analyses.

We observed no consistent evidence of an interaction between education and all other covariates, or residence location and all other covariates, after sensitivity analyses (Supplementary Figures S9, S10).

## Discussion

Participation in LTPA amongst adults was low in these sub-Saharan African populations when compared with participation in occupational physical activity and active travel. Our findings suggest that women, older adults, lower educated individuals, those who were self-employed, and rural residents were less likely to participate in LTPA, regardless of the physical activity undertaken in other domains. Few strong sociodemographic associations were observed for participation in occupational physical activity and active travel. With increasing urbanisation and mechanisation, concerted efforts should be made to ensure equitable access to LTPA and the associated health and social benefits.

Due to the multitude of methods for measuring and analysing participation in physical activity across its various domains, direct comparisons with the literature were limited [2, 39–41]. The observed prevalence of LTPA participation was slightly lower than the participation levels reported in the few studies conducted in SSA. Although no time frame was given, 25.4% of South African adults reported participating in some form of LTPA in a nationally-representative survey [42]. In Nigeria, 26.5% of a predominantly rural population participated in vigorous LTPA, and 20.4% participated in moderate LTPA at least once a week [43]. This was considerably lower than reported participation in HICs: on average 40% of Europeans were estimated to engage in regular LTPA per week [9].

Among the few studies conducted in African populations, evidence of a difference in participation in LTPA between the sexes was mixed [39, 43–45]. However, our findings are consistent with larger, more representative studies conducted in other low- and middle-income countries and HICs which report less participation in LTPA amongst women [46–48]. Possible explanations include constraints associated with women’s traditional responsibilities of managing childcare and domestic chores, [49] a lack of interest, [42] and a shortage of safe and accessible opportunities for LTPA [50].

The observed inverse relationship between age and participation in LTPA has previously been reported in African populations and globally [9, 39, 42, 45]. As people retire, and opportunities for physical activity at work or through travel potentially reduce, encouraging participation in LTPA through targeted and accessible programmes for older age groups may help prevent and manage non-communicable diseases (NCDs) and enable opportunities for socialising in a population which is often at risk of isolation.

We observed a strong positive association between education and participation in LTPA. Elsewhere in Africa, increased education has been associated with reduced overall physical activity, [51, 52] with low educational attainment associated with less participation in LTPA in high- and middle-income populations [9, 46]. Those with higher education may be more aware of the health and social benefits of LTPA and thus motivated to spend their leisure-time physically active, particularly if their work is sedentary [53]. It is also likely that a complex inter-relationship exists between education, employment and socioeconomic status (SES) [53]. Higher levels of educational attainment are likely to increase individuals’ opportunities for higher paid employment, reducing the financial constraints which may prevent participation in LTPA. Likewise, these jobs may enable regular participation in LTPA by providing greater flexibility and independence at work [54]. A number of studies globally have observed reduced participation in LTPA amongst those with lower SES [55]. In Kenya, 4.1–6.3% of individuals living in urban slums engaged in LTPA, compared with the national average of 23.4% [27, 39, 44]. Time and financial constraints, as well as inaptitude, have been reported as perceived barriers to participation for those with low SES in Botswana, South Africa and other HICs [42, 53, 56].

In these analyses, being self-employed was associated with lower participation in LTPA and increased likelihood of participating in occupational physical activity compared with those in private or public employment. In this context, self-employment is often characterised by low earnings and informal wages, with a high proportion involved in agricultural work [57]. This is consistent with findings from previous studies which observed that those in higher status occupations had higher levels of LTPA and lower levels of occupational physical activity compared with those in lower status occupations [54].

Rural participation in LTPA was notably lower compared with urban populations, while work and travel physical activity were notably higher, reflecting potential time and energy constraints on individuals. Rural populations are more likely to be involved in time-consuming agricultural work, which often requires high levels of energy expenditure, [52] thus these groups may not have the time or desire to participate in further physical activity during their limited leisure-time [42]. However, in these analyses RRs for participation in LTPA were not notably different after adjustment for participation and time spent physically active during work and travel. Thus, other factors may be associated with lower LTPA participation in rural populations, including fewer opportunities, especially in sparsely populated areas [58]. With increasing mechanisation, particularly in the agricultural sector, these individuals may be at risk of reduced occupational physical activity. Opportunities for LTPA should be available in order to maintain or increase physical activity levels in these populations.

BMI was inversely associated with participation in physical activity at work and through travel but no association was observed with LTPA in these populations. In many HICs, reductions in occupational physical activity have been associated with decreases in energy expenditure [2]. There is also evidence that although LTPA has increased, it has not been to sufficient levels to compensate for the reduction in occupational physical activity and consequently total physical activity has decreased markedly [59]. This is likely to have had an impact on weight maintenance and the risk of NCDs in these populations [60–62]. While there are likely to be other factors involved, relating to economic development and associated lifestyle changes including diet, [60, 62] creating opportunities to access the additional health and social benefits of LTPA and to compensate for reductions in occupational physical activity is undoubtedly an important consideration for public health officials in SSA, where the prevalence of overweight and obesity is already high [6–8, 63, 64].

To our knowledge this is the first meta-analysis using IPD from SSA to investigate participation within the three main domains of physical activity. Each study employed sampling strategies which aimed to recruit a representative sample of the national or subnational population from which they were drawn. The overall participant response was relatively high across studies. The studies included in these analyses all utilised or were consistent with the WHO STEPwise approach to non-communicable disease risk factor surveillance tool, a well-established survey tool for population surveillance which has been used in over 122 countries, [65] enabling the standardised collection of key variables. Data were collected by trained interviewers, with BMI objectively measured. Furthermore, identical methods were used to clean and analyse these data, enabling comparability of country-specific estimates and the calculation of more robust pooled estimates.

Self-report questionnaires are currently the main way to measure the domains of physical activity. While the reliability of the GPAQ is high, the validity tends to be low to moderate [66, 67]. However, the outcome of interest for validity studies tends to be volume of physical activity rather than domain, thus it is likely the validity of the GPAQ to measure domain participation is higher as participants are more likely to remember participating in physical activity within a certain domain than the exact time and intensity [66, 67].

While the studies included in these analyses aimed to be representative of their national or subnational populations, these findings may not be generalisable to other African populations or reflect the current situation for the populations of older studies. The small number of studies included in these analyses also limited the power to detect sources of heterogeneity at the study level. Due to the cross-sectional nature of these data the temporality of associations and seasonal variations of participation could not be assessed. Finally, there is the potential for residual confounding due to unaccounted for confounders.

In 2017, 60% of African countries had an operational policy or strategy which addressed physical activity, with limited evidence of their effectiveness [68]. To maintain or increase population physical activity levels it will be important for governments and health officials to identify the barriers and motivations associated with participation in LTPA, particularly amongst those groups where participation is notably lower. Providing free recreational facilities will not automatically increase participation, as observed in Kenya [44]: instead a whole system approach is required [5]. Mass media campaigns, such as ‘This Girl Can’ in the UK and ‘Kau Mai Tonga’ in Tonga, have shown success in empowering women to engage in physical activity [11, 69, 70]. Such campaigns are a recommended NCD ‘Best Buy’ and can help shift social norms and perceived barriers to participation if designed appropriately for the African context [71]. Likewise, improving access to opportunities for physical activity throughout life will be critical to ensuring that participation in LTPA does not decline with age. Exposure to physical activity in school was an important motivator for adult women who participated in LTPA in South Africa [42]. Furthermore, positive experiences of physical activity at school and in other settings is important for building individual’s confidence to participate [11, 53]. Along with the health benefits, the opportunity to socialise through LTPA was a key motivator for older people in South Africa [42]. Tailoring physical activity programmes to meet the diverse needs and motivations of groups who are currently at risk of lower participation in LTPA will be important for encouraging and maintaining participation [5]. Likewise, investing in accessible and safe environments for physical activity will be critical for achieving equitable participation in LTPA [50, 72–74].

Furthermore, concerted efforts are needed to strengthen the surveillance infrastructure within SSA to collect longitudinal data on the physical activity patterns of these populations, ideally using objective measurement tools supplemented with diaries, to capture the domain in which physical activity is undertaken [75, 76]. This is important as recent studies suggest differential health benefits across physical activity domains [6–8]. Additionally, high levels of occupational physical activity have been associated with increased risk of all-cause mortality and cardiovascular events, particularly in men, [7, 77] with one study reporting that LTPA may modify these associations [78]. However, further studies are needed to understand the specific mechanisms by which these associations may be explained and to better account for residual confounding [75, 79]. Considering the high levels of occupational physical activity, the low participation in LTPA, and the increasing burden of NCDs among populations in SSA, these associations should be of particular interest to governments; however, without prospective research, associations between domains of physical activity and health outcomes cannot be explored. Finally, such surveillance platforms would enable the monitoring of trends and the evaluation of interventions to more clearly establish their effectiveness and inform scale-up strategies [79].

## Conclusions

This study adds to the limited evidence on LTPA participation amongst adults in SSA. We show that participation in LTPA is low in these populations, with women, older adults, less educated, self-employed and rural populations less likely to participate in LTPA. Further research is necessary to understand the personal, social and environmental barriers and motivators for participation in LTPA in these groups. Likewise, investment in surveillance infrastructure will allow us to track temporal trends in domain participation and improve the evidence base for policy development. Finally, as increasing urbanisation and mechanisation leads to declines in population physical activity levels, political and financial commitment from governments is necessary to ensure that the health and social benefits of LTPA are accessible to all [80].

## Supplementary information

**Additional file 1.**

## Data Availability

The data that support the findings of this study are available from the corresponding author upon reasonable request.

## Abbreviations

BMI: Body-mass index
DRC: Democratic Republic of Congo
WHO: World Health Organization
STEPS: WHO STEPwise approach to non-communicable disease risk factor surveillance tool
GPAQ: Global Physical Activity Questionnaire
HICs: High-income countries
IPD: Individual participant data
LTPA: Leisure-time physical activity
SSA: Sub-Saharan Africa
RR: Risk ratio
MET: Metabolic equivalent
NCD: Non-communicable disease
SES: Socio-economic status
