# Supplementary material for: Sociodemographic inequities associated with participation in leisure-time physical activity in sub-Saharan Africa: an individual participant data meta-analysis
Source: BMC Public Health. 2020 Jun 15;20:927. doi: 10.1186/s12889-020-08987-w (PMC7296740; doi:10.1186/s12889-020-08987-w)
Supplement: Supplementary file 1 — Additional file 1. [file 12889_2020_8987_MOESM1_ESM.docx]

**Supplementary methods:**

**Education categorisation**

Education levels were measured slightly differently between surveys, depending on the individual country’s education system. Therefore, education level was grouped into four categories based on the highest education level completed: primary education not completed, primary education completed, secondary education completed and tertiary education completed Typically, those who had not completed primary education had either no formal schooling or had not completed primary school. Those who had completed primary education had completed primary school or had not completed secondary school or in the case of Seychelles, had not completed obligatory school (up to the age of 15-16 years). Those who had completed secondary education had completed secondary school or high school but had not completed tertiary education. While those who had completed tertiary education had either completed a university or college degree, or a further post-graduate degree. Thus, the typical ages of students in each category may differ.

**Employment categorisation**

Employment status was re-categorised from nine to four categories: government employees and non-government employees were coded as ‘public or private employees’; self-employed remained a distinct category; non-paid, home-makers and students were grouped as ‘non-income workers’; and retired and unemployed individuals (both those able and unable to work) were categorised as having ‘no occupation’. South Africa and Togo each included an additional employment category, part-time employment and farmer respectively, which was not easily re-categorised and therefore these individuals were excluded from the main analyses. Including farmers within the self-employed category for Togo and part-time workers within the unemployed category for South Africa did not notably change the RRs for all sociodemographic characteristics (data not shown).

**Supplementary Tables and Figures:**

**Table S1**. Level of clustering for each dataset used in analyses and response for each country

| **Study** |  | **Sampling unit** | | | | | | | |  | **Response** |
| --- | --- | --- | --- | --- | --- | --- | --- | --- | --- | --- | --- |
|  |  | **Primary** | |  | **Secondary** | |  | **Tertiary** | |  |  |
|  |  | Cluster level | N |  | Cluster level | N |  | Cluster level | N |  | % |
| DRC |  | Stratum | 9 |  | Quarter | 40 |  | Household | 765 |  | 77.0% |
| Guinea |  | Stratum | 2 |  | Cluster | 63 |  |  |  |  | 98.8% |
| Kenya |  | County | 47 |  | Cluster | 200 |  |  |  |  | 79.2% |
| Liberia |  | County | 5 |  | Clans | 118 |  |  |  |  | 60.3% |
| Seychelles |  | Island | 3 |  | District | 27 |  |  |  |  | 72.9% |
| South Africa |  | Planning unit cluster | 9 |  | Household | 817 |  |  |  |  | 92.6% |
| Tanzania |  | District | 50 |  | EA | 246 |  |  |  |  | 94.7% |
| Zanzibar |  | Stratum | 2 |  | Shehia | 201 |  |  |  |  | 98.2% |
| Togo |  | Health region | 6 |  | EA | 298 |  |  |  |  | 91.3% |
| Uganda |  | EA | 349 |  |  |  |  |  |  |  | 81.4% |

*N: number of clusters; EA: enumeration area; Response: percentage of the participants who enrolled in the survey from the target sample size.*

**Table S2.** Adjusted distribution of sociodemographic characteristics across studies

|  |  | **DRC** | **Guinea** | **Kenya** | **Liberia** | **Seychelles** | **South Africa** | **Tanzania** | **Zanzibar** | **Togo** | **Uganda** |
| --- | --- | --- | --- | --- | --- | --- | --- | --- | --- | --- | --- |
|  |  | **N = 1502** | **N = 2125** | **N = 4184** | **N = 2206** | **N = 1232** | **N = 1014** | **N = 5525** | **N = 2640** | **N = 2051** | **N = 3543** |
|  |  | % | % | % | % | % | % | % | % | % | % |
|  |  | (95%CI) | (95%CI) | (95%CI) | (95%CI) | (95%CI) | (95%CI) | (95%CI) | (95%CI) | (95%CI) | (95%CI) |
| **Sex^1^** |  |  |  |  |  |  |  |  |  |  |  |
| Men |  | 38.7 | 46.4 | 41.1 | 40.9 | 42.8 | 27.0 | 45.8 | 38.3 | 37.6 | 42.1 |
|  |  | (36.8, 40.7) | (46.3, 46.5) | (38.6, 43.6) | (34.0, 47.8) | (42.6, 43.0) | (22.1, 31.9) | (43.2, 48.4) | (37.4, 39.2) | (32.0, 43.1) | (40.3, 43.8) |
| Women |  | 61.3 | 53.6 | 58.7 | 57.4 | 57.2 | 72.5 | 53.5 | 61.7 | 61.9 | 57.9 |
|  |  | (59.3, 63.2) | (53.5, 53.7) | (56.3, 61.0) | (53.8, 61.0) | (57.1, 57.4) | (68.6, 76.4) | (50.9, 56.1) | (60.8, 62.6) | (56.4, 67.3) | (56.2, 59.7) |
| **Age (years)^2^** |  |  |  |  |  |  |  |  |  |  |  |
| 18-24 |  | 26.2 | 22.4 | 17.0 | 1.9 | N/A | 24.8 | 0.8 | 0.7 | 25.9 | 22.6 |
|  |  | (24.5, 27.9) | (12.2, 32.7) | (15.4, 18.7) | (0.4, 3.4) |  | (20.7, 28.9) | (0.4, 1.2) | (0.6, 0.8) | (22.3, 29.5) | (21.2, 24.0) |
| 25-34 |  | 30.9 | 29.2 | 29.7 | 39.6 | 21.9 | 20.8 | 31.2 | 33.1 | 32.7 | 29.8 |
|  |  | (28.1, 33.7) | (26.5, 31.9) | (27.7, 31.7) | (30.7, 48.5) | (21.3, 22.5) | (16.6, 25.0) | (28.8, 33.5) | (32.1, 34.0) | (30.3, 35.1) | (28.2, 31.4) |
| 35-44 |  | 17.1 | 19.1 | 23.2 | 28.3 | 22.5 | 15.2 | 29.2 | 28.2 | 20.4* | 21.6 |
|  |  | (15.6, 18.6) | (13.6, 24.6) | (21.5, 24.9) | (24.6, 32.0) | (22.1, 22.9) | (12.9, 17.5) | (27.4, 31.0) | (24.4, 32.0) | (18.5, 22.3) | (20.2, 23.0) |
| 45-54 |  | 10.0 | 16.3 | 13.8 | 15.8 | 26.9 | 15.6 | 21.8 | 23.7 | 13.9 | 14.6 |
|  |  | (7.3, 12.8) | (13.0, 19.6) | (12.4, 15.3) | (10.2, 21.4) | (26.0, 27.9) | (14.6, 16.6) | (20.4, 23.2) | (22.8, 24.5) | (12.5, 15.4) | (13.2, 16.1) |
| 55-64 |  | 9.1 | 11.1 | 10.2 | 10.2 | 28.7 | 12.8 | 14.7 | 14.4 | 7.1 | 7.8 |
|  |  | (7.5, 10.6) | (8.4, 13.8) | (8.7, 11.6) | (3.5, 16.9) | (27.9, 29.4) | (9.1, 16.4) | (13.0, 16.4) | (11.0, 17.7) | (5.7, 8.5) | (6.6, 8.9) |
| 65+ |  | 5.7 | 0.1 | 3.8 | N/A | N/A | 9.0 | 0.1 | N/A | N/A | 3.0 |
|  |  | (4.1, 7.2) | (0.1, 0.1) | (2.8, 4.7) |  |  | (6.8, 11.3) | (0.0, 0.1) |  |  | (2.2, 3.8) |
| **Education** |  |  |  |  |  |  |  |  |  |  |  |
| No primary |  | 14.3 | 56.3 | 35.7 | 47.9 | N/A | 1.0 | 28.2 | 47.4 | 48.9 | 14.3 |
|  |  | (9.5, 19.2) | (32.7, 79.9) | (30.6, 40.8) | (31.2, 64.7) |  | (0.1, 1.9) | (24.9, 31.4) | (14.8, 80.0) | (36.9, 61.0) | (12.7, 16.0) |
| Primary |  | 49.7 | 8.6 | 28.6 | 10.8 | 10.2 | 21.0 | 58.6 | 21.2 | 22.5 | 41.0 |
|  |  | (46.6, 52.7) | (6.4, 10.9) | (23.9, 33.2) | (6.0, 15.6) | (9.4, 10.9) | (17.4, 24.6) | (55.5, 61.7) | (11.5, 31.0) | (20.1, 24.9) | (38.9, 43.1) |
| Secondary |  | 20.8 | 18.7 | 14.2 | 28.2 | 84.2 | 71.4 | 5.6 | 22.3 | 20.0 | 32.7 |
|  |  | (17.8, 23.8) | (1.5, 35.9) | (11.7, 16.7) | (16.3, 40.1) | (82.6, 85.8) | (69.0, 73.8) | (4.3, 6.9) | (4.8, 39.7) | (12.1, 27.9) | (30.8, 34.6) |
|  | | | |  |  |  |  |  |  |  |  |
| *(Education continued from previous page)* | | | |  |  |  |  |  |  |  |  |
| Tertiary |  | 10.2 | 6.8 | 7.6 | 2.0 | 3.5 | 3.5 | 1.8 | 1.0 | 2.1 | 5.4 |
|  |  | (5.5, 14.9) | (-6.6, 20.1) | (5.9, 9.3) | (-0.6, 4.7) | (-0.2, 9.1) | (2.0, 5.1) | (1.2, 2.5) | (-1.3, 3.3) | (0.6, 3.7) | (4.1, 6.6) |
| **Employment** |  |  |  |  |  |  |  |  |  |  |  |
| Private or public employee |  | 15.0 | 10.5 | 13.9 | 13.4 | 68.9 | 20.5 | 6.0 | 11.7 | 10.3 | 6.8 |
|  |  | (13.2, 16.9) | (-5.0, 26.0) | (11.3, 16.4) | (9.3, 17.5) | (64.4, 73.4) | (18.2, 22.8) | (4.7, 7.3) | (1.1, 22.3) | (7.8, 12.8) | (5.5, 8.2) |
| Self-employed |  | 25.0 | 42.1 | 36.7 | 53.1 | 20.1 | 8.0 | 69.6 | 47.0 | 37.8 | 54.8 |
|  |  | (22.1, 27.9) | (23.5, 60.7) | (32.1, 41.3) | (37.2, 68.9) | (12.1, 28.1) | (6.1, 9.9) | (66.1, 73.2) | (38.4, 55.7) | (25.0, 50.5) | (51.6, 58.0) |
| Non-income worker |  | 44.3 | 33.6 | 25.9 | 14.9 | 3.2‡ | 13.1 | 16.9 | 33.6 | 40.2 | 24.0 |
|  |  | (40.6, 47.9) | (25.2, 42.0) | (22.6, 29.3) | (3.6, 26.2) | (2.1, 4.2) | (11.1, 15.1) | (14.2, 19.6) | (27.5, 39.7) | (28.5, 51.8) | (21.4, 26.5) |
| No occupation |  | 13.9 | 4.9 | 8.4 | 7.2 | 11.3 | 58.4 | 1.6 | 3.2 | 4.8 | 3.9 |
|  |  | (11.2, 16.6) | (0.3, 9.5) | (6.3, 10.5) | (-0.4, 14.8) | (10.3, 12.3) | (55.7, 61.1) | (1.1, 2.2) | (-1.3, 7.7) | (3.0, 6.6) | (3.0, 4.9) |
| **BMI** |  |  |  |  |  |  |  |  |  |  |  |
| Underweight |  | 17.6 | 11.5 | 9.6 | 4.1 | 2.0 | 2.9 | 11.1 | 9.1 | 7.3 | 8.3 |
|  |  | (14.6, 20.6) | (9.8, 13.2) | (7.7, 11.6) | (1.8, 6.5) | (1.9, 2.2) | (2.0, 3.8) | (9.5, 12.7) | (0.7, 17.4) | (5.4, 9.2) | (7.1, 9.5) |
| Healthy weight |  | 59.5 | 61.8 | 55.8 | 46.6 | 30.2 | 35.4 | 62.3 | 53.1 | 63.7 | 68.3 |
|  |  | (56.6, 62.3) | (54.0, 69.5) | (53.3, 58.3) | (37.7, 55.6) | (27.9, 32.5) | (32.7, 38.1) | (59.3, 65.2) | (42.9, 63.2) | (56.0, 71.4) | (66.6, 70.0) |
| Overweight |  | 14.6 | 17.4 | 20.4 | 28.4 | 35.2 | 23.7 | 15.7 | 21.9 | 19.4 | 16.0 |
|  |  | (10.6, 18.6) | (12.3, 22.5) | (18.2, 22.6) | (26.4, 30.3) | (33.4, 37.0) | (21.2, 26.2) | (13.8, 17.6) | (15.9, 27.9) | (15.1, 23.8) | (14.5, 17.6) |
| Obese |  | 5.8 | 6.9 | 8.8 | 16.6 | 32.5 | 38.1 | 5.9 | 12.3 | 7.6 | 5.2 |
|  |  | (4.6, 7.0) | (-0.4, 14.3) | (7.0, 10.6) | (8.3, 24.8) | (32.0, 33.1) | (36.8, 39.3) | (4.3, 7.4) | (0.7, 24.0) | (3.8, 11.5) | (4.1, 6.3) |
| **Physical activity** | |  |  |  |  |  |  |  |  |  |  |
| Insufficient physical activity |  | 23.0 | 13.2 | 4.8 | 15.1 | 14.0 | 7.8 | 3.5 | 9.4 | 8.0 | 4.0 |
|  |  | (19.5, 26.6) | (1.7, 24.6) | (3.3, 6.3) | (6.7, 23.5) | (3.3, 24.7) | (4.5, 11.0) | (2.5, 4.5) | (-0.1, 18.9) | (5.6, 10.4) | (3.0, 4.9) |
| Participation in physical activity |  | 80.8 | 92.7 | 96.3 | 85.5 | 94.7 | 97.7 | 97.6 | 95.9 | 94.9 | 97.1 |
|  |  | (74.9, 86.7) | (89.5, 96.0) | (94.7, 97.8) | (77.1, 93.8) | (93.9, 95.5) | (96.4, 99.1) | (96.7, 98.5) | (92.2, 99.6) | (93.1, 96.7) | (96.5, 97.7) |
| **Residence location***†^3^* | |  |  |  |  |  |  |  |  |  |  |
| Urban |  | N/A | 67.2 | 46.8 | N/A | N/A | N/A | 24.1 | 32.7 | 39.8 | 26.5 |
|  |  |  | (65.2, 69.2) | (45.3, 48.3) |  |  |  | (22.9, 25.2) | (30.9, 34.5) | (37.7, 42.0) | (25.0, 27.9) |
| Rural |  | N/A | 32.8 | 53.2 | N/A | N/A | N/A | 75.9 | 67.3 | 60.2 | 73.5 |
|  |  |  | (30.8, 34.8) | (51.7, 54.7) |  |  |  | (74.8, 77.1) | (65.5, 69.1) | (58.0, 62.3) | (72.1, 75.0) |

*†: Kenya: N = 4138*

*%: Adjusted prevalence estimates, based on mixed effects Poisson regression models with robust standard errors, adjusted for sex, age and clustering; 95%CI: 95% confidence interval; N/A: data not collected; *: The primary sampling unit for the adjusted prevalence was not included to facilitate model convergence; ‡: Not adjusted for sex as there were no male non-income workers; BMI: body-mass index; Underweight (BMI: <18.5kg/m^2^); Healthy weight (BMI: 18.5–24.9kg/m^2^); Overweight (BMI: 25.0–29.9kg/m^2^); Obese (BMI:* $\geq$*30.0kg/m^2^).*

*^1^ Adjusted prevalence estimates for sex are based on mixed effects Poisson regression models with robust standard errors, adjusted for age and clustering.*

*^2^ Adjusted prevalence estimates for age group are based on mixed effects Poisson regression models with robust standard errors, adjusted for sex and clustering.*

*^3^ Adjusted prevalence estimates for age group are based on mixed effects Poisson regression models with robust standard errors, adjusted for sex and age.*

*Adjusted prevalence estimates may not add up to 100% due to the effects of adjustment.*

**Table S3.** Adjusted prevalence of participation in physical activity through leisure-time physical activity

|  |  | **DRC** | **Guinea** | **Kenya** | **Liberia** | **Seychelles** | **South Africa** | **Tanzania** | **Zanzibar** | **Togo** | **Uganda** |
| --- | --- | --- | --- | --- | --- | --- | --- | --- | --- | --- | --- |
|  |  | **N = 1502** | **N = 2125** | **N = 4184** | **N = 2206** | **N = 1232** | **N = 1014** | **N = 5525** | **N = 2640** | **N = 2051** | **N = 3543** |
|  |  | %  (95%CI) | %  (95%CI) | %  (95%CI) | %  (95%CI) | %  (95%CI) | %  (95%CI) | %  (95%CI) | %  (95%CI) | %  (95%CI) | %  (95%CI) |
| **Overall** |  | 1.6 | 15.9 | 18.4 | 20.3 | 25.3 | 25.0 | 27.1 | 19.4 | 23.4 | 22.7 |
|  |  | (0.7, 2.4) | (10.5, 21.4) | (15.2, 21.6) | (14.9, 25.6) | (23.6, 27.0) | (20.5, 29.4) | (24.7, 29.4) | (17.7, 21.0) | (18.0, 28.8) | (20.6, 24.8) |
| **Sex ^1^** |  |  |  |  |  |  |  |  |  |  |  |
| Men |  | 2.8 | 28.8 | 29.3 | 30.7 | 30.0 | 48.4 | 34.9 | 38.7 | 45.2 | 33.9 |
|  |  | (1.3, 4.3) | (19.6, 38.0) | (25.3, 33.2) | (21.2, 40.3) | (27.0, 32.9) | (42.1, 54.7) | (31.8, 38.0) | (27.2, 50.3) | (39.9, 50.4) | (31.0, 36.7) |
| Women |  | 0.8 | 4.1 | 11.2 | 12.3 | 21.8 | 14.8 | 20.9 | 5.1 | 9.3 | 14.7 |
|  |  | (0.0, 1.6) | (2.2, 6.0) | (8.0, 14.5) | (6.6, 18.1) | (21.0, 22.7) | (10.4, 19.2) | (18.9, 23.0) | (-0.6, 10.8) | (4.6, 13.9) | (12.2, 17.2) |
| **Age (years) ^2^** |  |  |  |  |  |  |  |  |  |  |  |
| 18-24 |  | 2.4 | 29.2 | 36.3 | 29.7 | N/A | 39.0 | 41.9 | 42.1 | 45.2 | 39.1 |
|  |  | (0.5, 4.4) | (28.5, 29.8) | (31.9, 40.7) | (25.1, 34.3) |  | (34.9, 43.1) | (31.4, 52.5) | (37.3, 46.9) | (40.2, 50.2) | (35.6, 42.5) |
| 25-34 |  | 0.7 | 18.5 | 20.4 | 25.9 | 31.4 | 25.4 | 34.2 | 25.4 | 22.0 | 25.9 |
|  |  | (-0.4, 1.7) | (6.0, 30.9) | (15.6, 25.2) | (17.8, 34.0) | (11.5, 51.2) | (16.5, 34.3) | (31.6, 36.9) | (22.5, 28.4) | (13.5, 30.5) | (23.0, 28.9) |
| 35-44 |  | 0.6 | 8.4 | 17.6 | 18.7 | 29.6 | 22.1 | 27.3 | 16.3 | 17.0 | 18.2 |
|  |  | (-1.1, 2.3) | (6.3, 10.6) | (13.1, 22.0) | (11.7, 25.7) | (27.5, 31.6) | (14.8, 29.4) | (24.1, 30.6) | (15.1, 17.5) | (11.4, 22.7) | (15.3, 21.1) |
| 45-54 |  | 2.2 | 9.8 | 10.6 | 10.3 | 23.2 | 18.6 | 25.1 | 16.9 | 18.5 | 15.1 |
|  |  | (-0.8, 5.3) | (7.2, 12.4) | (6.9, 14.3) | (8.0, 12.5) | (20.2, 26.2) | (11.5, 25.7) | (22.3, 27.9) | (13.4, 20.4) | (10.3, 26.6) | (10.4, 19.8) |
| 55-64 |  | 0.7* | 4.8 | 8.4 | 10.2 | 22.1 | 15.9 | 17.5 | 14.7 | 15.8 | 12.0 |
|  |  | (-1.2, 2.5) | (-5.1, 14.6) | (4.3, 12.5) | (8.4, 12.0) | (21.6, 22.6) | (3.1, 28.7) | (13.8, 21.3) | (10.8, 18.6) | (8.8, 22.7) | (8.2, 15.8) |
| 65+ |  | 0.4* | 0.0 | 6.4 | N/A | N/A | 5.2 | 66.7‡ | N/A | N/A | 5.2 |
|  |  | (-1.0, 1.8) | (0.0, 0.0) | (0.8, 12.0) |  |  | (-2.5, 12.9) | (1.3, 132.0) |  |  | (-0.7, 11.1) |
| **Education** |  |  |  |  |  |  |  |  |  |  |  |
| No primary |  | 0.7 | 6.5 | 8.3 | 10.9 | N/A | 27.3 | 21.3 | 13.4 | 7.3 | 11.6 |
|  |  | (-0.1, 1.5) | (4.5, 8.5) | (5.4, 11.3) | (6.8, 15.0) |  | (1.0, 53.6) | (18.0, 24.6) | (11.0, 15.7) | (1.4, 13.2) | (8.0, 15.3) |
| Primary |  | 1.3 | 18.8 | 21.4 | 24.1 | 9.4 | 11.2 | 27.8 | 22.3 | 31.0 | 17.7 |
|  |  | (-0.6, 3.1) | (11.2, 26.4) | (17.5, 25.3) | (12.9, 35.3) | (7.2, 11.7) | (4.1, 18.2) | (24.9, 30.7) | (13.4, 31.2) | (22.5, 39.5) | (14.8, 20.5) |
| Secondary |  | 3.5 | 32.4 | 32.8 | 25.5 | 25.8 | 28.0 | 43.0 | 26.4 | 51.2 | 31.1 |
|  |  | (1.6, 5.3) | (31.8, 33.0) | (27.4, 38.2) | (21.7, 29.2) | (23.4, 28.3) | (22.6, 33.4) | (38.2, 47.8) | (24.5, 28.3) | (46.1, 56.2) | (28.3, 33.8) |
| Tertiary |  | 1.4 | 34.5 | 38.2 | 33.0 | 47.1 | 33.3 | 41.8 | 38.0 | 44.7 | 41.5 |
|  |  | (-0.8, 3.6) | (32.2, 36.8) | (32.6, 43.9) | (27.4, 38.5) | (44.6, 49.5) | (29.3, 37.4) | (34.4, 49.2) | (33.1, 43.0) | (39.6, 49.8) | (36.3, 46.8) |
| **Employment** |  |  |  |  |  |  |  |  |  |  |  |
| Private or public employee |  | 2.5 | 27.1 | 29.3 | 27.8 | 26.8 | 29.3 | 40.7 | 32.9 | 40.4 | 33.3 |
|  |  | (0.6, 4.4) | (23.6, 30.7) | (24.1, 34.6) | (24.5, 31.0) | (24.3, 29.4) | (21.6, 37.0) | (36.3, 45.1) | (25.9, 40.0) | (36.5, 44.4) | (28.3, 38.4) |
| Self-employed |  | 1.1 | 11.2 | 16.0 | 18.1 | 24.4 | 27.2 | 27.8 | 22.8 | 17.4 | 22.0 |
|  |  | (0.2, 2.1) | (5.1, 17.3) | (12.4, 19.6) | (10.6, 25.7) | (21.5, 27.3) | (22.5, 31.8) | (25.4, 30.2) | (14.0, 31.6) | (10.3, 24.4) | (19.2, 24.9) |
| Non-income worker |  | 1.3 | 20.0 | 17.1 | 18.5 | 20.5 | 43.6 | 20.2 | 6.6 | 29.8 | 24.1 |
|  |  | (-1.0, 3.6) | (19.6, 20.4) | (12.7, 21.4) | (15.4, 21.6) | (12.6, 28.4) | (36.3, 50.9) | (16.5, 23.9) | (3.9, 9.2) | (25.0, 34.6) | (21.4, 26.8) |
| No occupation |  | 2.6 | 10.7* | 18.9 | 20.4 | 18.0 | 18.1 | 24.6 | 12.5 | 24.1 | 12.9 |
|  |  | (-0.2, 5.4) | (-0.3 21.6) | (13.5, 24.4) | (17.6, 23.1) | (15.7, 20.2) | (13.6, 22.5) | (16.4, 32.8) | (-22.1, 47.2) | (18.5, 29.7) | (8.4, 17.4) |
| **BMI** |  |  |  |  |  |  |  |  |  |  |  |
| Underweight |  | 1.8 | 14.8 | 17.8 | 19.4 | 16.5 | 31.1 | 22.3 | 11.0 | 28.2 | 18.0 |
|  |  | (0.3, 3.2) | (12.7, 17.0) | (13.1, 22.5) | (14.4, 24.4) | (-5.7, 38.6) | (19.1, 43.0) | (17.2, 27.4) | (10.8, 11.2) | (17.9, 38.4) | (13.8, 22.2) |
| Healthy weight |  | 2.0 | 18.5 | 19.8 | 23.5 | 31.7 | 34.3 | 29.4 | 22.0 | 28.1 | 24.8 |
|  |  | (1.0, 3.1) | (12.7, 24.3) | (15.8, 23.9) | (18.0, 28.9) | (13.9, 49.5) | (29.7, 38.8) | (26.9, 31.9) | (17.9, 26.1) | (22.0, 34.1) | (22.4, 27.1) |
| Overweight |  | 1.4 | 13.7 | 18.5 | 18.8 | 26.0 | 24.9 | 25.7 | 19.8 | 21.4 | 24.4 |
|  |  | (0.1, 2.6) | (10.4, 17.0) | (14.1, 22.9) | (9.0, 28.6) | (24.1, 28.0) | (17.0, 32.7) | (22.5, 28.9) | (17.2, 22.3) | (15.9, 26.9) | (20.8, 27.9) |
| Obese |  | 2.2* | 7.5 | 15.7 | 12.7 | 21.4 | 14.9 | 26.6 | 14.9 | 17.1 | 22.4 |
|  |  | (-0.8, 5.2) | (3.7, 11.3) | (10.7, 20.7) | (9.7, 15.6) | (19.9, 23.0) | (9.4, 20.5) | (23.0, 30.3) | (12.6, 17.1) | (11.6, 22.6) | (16.8, 28.1) |
| **Residence location***†* | |  |  |  |  |  |  |  |  |  |  |
| Urban |  | N/A | 19.2 | 22.9 | N/A | N/A | N/A | 27.3 | 21.4* | 32.1 | 30.2 |
|  |  |  | (17.2, 21.3) | (19.1, 26.6) |  |  |  | (22.8, 31.8) | (17.8, 25.1) | (26.5, 37.6) | (26.6, 33.7) |
| Rural |  | N/A | 9.3* | 14.3 | N/A | N/A | N/A | 26.9 | 17.8 | 18.1 | 19.8 |
|  |  |  | (5.8, 12.8) | (10.3, 18.4) |  |  |  | (24.2, 29.7) | (12.8, 22.8) | (13.8, 22.4) | (17.4, 22.3) |

*† Kenya: N = 4138.*

*%: Adjusted prevalence based on mixed effects Poisson regression models with robust standard errors, adjusted for sex, age and clustering; 95%CI: 95% confidence interval; N/A: data not collected;* **: The primary sampling unit for the adjusted prevalence was not included to facilitate model convergence;* ‡: *The primary and secondary sampling unit for the adjusted prevalence was not included to facilitate model convergence and sex was not included as only male; BMI: body-mass index; Underweight (BMI: <18.5kg/m^2^); Healthy weight (BMI: 18.5–24.9kg/m^2^); Overweight (BMI: 25.0–29.9kg/m^2^); Obese (BMI:* $\geq$*30.0kg/m^2^).*

*^1^ Adjusted prevalence estimates for sex are based on mixed effects Poisson regression models with robust standard errors, adjusted for age and clustering.*

*^2^ Adjusted prevalence estimates for age group are based on mixed effects Poisson regression models with robust standard errors, adjusted for sex and clustering.*

*Adjusted prevalence estimates may not add up to 100% due to the effects of adjustment.*

**Table S4.** Adjusted prevalence of participation in occupational physical activity

|  |  | **DRC** | **Guinea** | **Kenya** | **Liberia** | **Seychelles** | **South Africa** | **Tanzania** | **Zanzibar** | **Togo** | **Uganda** |
| --- | --- | --- | --- | --- | --- | --- | --- | --- | --- | --- | --- |
|  |  | **N = 1502** | **N = 2125** | **N = 4184** | **N = 2206** | **N = 1232** | **N = 1014** | **N = 5525** | **N = 2640** | **N = 2051** | **N = 3543** |
|  |  | %  (95%CI) | %  (95%CI) | %  (95%CI) | %  (95%CI) | %  (95%CI) | %  (95%CI) | %  (95%CI) | %  (95%CI) | %  (95%CI) | %  (95%CI) |
| **Overall** |  | 4.0 | 49.6 | 82.5 | 70.7 | 77.1 | 88.4 | 92.0 | 76.3 | 63.9 | 89.4 |
|  |  | (1.6, 6.4) | (35.7, 63.5) | (76.2, 88.8) | (51.2, 90.2) | (74.1, 80.1) | (82.7, 94.0) | (89.7, 94.3) | (50.6, 102.1) | (54.5, 73.4) | (87.9, 90.8) |
| **Sex^1^** |  |  |  |  |  |  |  |  |  |  |  |
| Men |  | 7.0 | 58.7 | 85.3 | 72.5 | 76.3 | 91.0 | 90.7 | 84.6 | 63.4 | 90.1 |
|  |  | (3.6, 10.4) | (42.1, 75.3) | (81.4, 89.2) | (52.8, 92.1) | (73.2, 79.3) | (86.8, 95.3) | (88.3, 93.0) | (65.0, 104.2) | (57.7, 69.2) | (88.2, 91.9) |
| Women |  | 2.3 | 43.0 | 82.2 | 69.1 | 77.7 | 87.4 | 93.1 | 71.0 | 66.5 | 88.8 |
|  |  | (0.2, 4.3) | (31.3, 54.7) | (74.8, 89.5) | (53.6, 84.7) | (74.7, 80.8) | (81.1, 93.6) | (90.6, 95.7) | (41.6, 100.5) | (57.5, 75.5) | (87.0, 90.7) |
| **Age (years)^2^** |  |  |  |  |  |  |  |  |  |  |  |
| 18-24 |  | 4.3 | 52.9 | 79.0 | 47.3 | N/A | 87.8 | 95.2 | 57.9 | 64.2 | 88.8 |
|  |  | (-1.3, 9.9) | (37.1, 68.6) | (71.4, 86.6) | (41.6, 52.9) |  | (84.1, 91.5) | (89.8, 100.6) | (30.9, 84.9) | (53.7, 74.6) | (86.1, 91.4) |
| 25-34 |  | 3.5 | 50.1 | 85.9 | 68.8 | 74.4 | 90.8 | 92.9 | 77.8 | 68.0 | 88.9 |
|  |  | (1.6, 5.5) | (37.7, 62.6) | (81.4, 90.4) | (52.2, 85.5) | (73.5, 75.4) | (83.9, 97.8) | (90.5, 95.2) | (50.9, 104.6) | (59.7, 76.2) | (86.8, 91.1) |
| 35-44 |  | 4.6 | 58.2 | 88.5 | 72.0 | 80.1 | 91.6 | 93.7 | 78.3 | 66.2 | 90.8 |
|  |  | (-0.9, 10.1) | (38.2, 78.1) | (84.7, 92.4) | (52.8, 91.1) | (73.4, 86.9) | (85.1, 98.0) | (91.1, 96.3) | (50.7, 105.9) | (56.3, 76.1) | (88.5, 93.2) |
| 45-54 |  | 4.4 | 50.7 | 87.7* | 67.0 | 77.1 | 89.9 | 91.9 | 77.9 | 69.2 | 91.2 |
|  |  | (1.9, 7.0) | (31.9, 69.5) | (84.3, 91.0) | (49.3, 84.7) | (70.7, 83.5) | (80.8, 99.0) | (89.6, 94.2) | (55.5, 100.3) | (60.8, 77.7) | (88.6, 93.9) |
| 55-64 |  | 3.1 | 43.5 | 81.4 | 63.8 | 76.8 | 88.6 | 87.0 | 66.1 | 54.1 | 84.7 |
|  |  | (-2.3, 8.4) | (20.8, 66.2) | (73.5, 89.2) | (45.1, 82.6) | (74.9, 78.7) | (83.1, 94.2) | (83.4, 90.5) | (40.7, 91.6) | (42.3, 65.9) | (80.2, 89.3) |
| 65+ |  | 5.7* | 0.0 | 73.4 | N/A | N/A | 76.0 | 100.0‡ | N/A | N/A | 90.0 |
|  |  | (1.2, 10.3) | (0.0, 0.0) | (63.7, 83.1) |  |  | (65.7, 86.4) | (100.0, 100.0) |  |  | (84.7, 95.4) |
| **Education** |  |  |  |  |  |  |  |  |  |  |  |
| No primary |  | 2.6 | 48.1 | 83.5 | 69.2 | N/A | 90.9 | 91.7 | 79.4 | 65.1 | 91.6 |
|  |  | (-1.9, 7.1) | (30.6, 65.6) | (76.6, 90.5) | (54.1, 84.3) |  | (79.4, 102.4) | (89.2, 94.2) | (58.9, 100.0) | (55.2, 75.1) | (89.0, 94.2) |
| Primary |  | 4.9 | 57.0 | 88.0 | 73.1 | 78.0 | 84.5 | 93.5 | 76.9 | 69.8 | 93.3 |
|  |  | (2.2, 7.7) | (47.8, 66.2) | (84.8, 91.2) | (53.8, 92.4) | (74.1, 81.8) | (75.8, 93.2) | (91.3, 95.7) | (52.2, 101.6) | (61.3, 78.4) | (91.7, 94.8) |
| Secondary |  | 3.3 | 49.3 | 85.3 | 68.9 | 78.0 | 89.1 | 85.5 | 72.2 | 63.8 | 87.1 |
|  |  | (-0.8, 7.5) | (41.7, 56.8) | (81.2, 89.5) | (46.6, 91.1) | (74.9, 81.1) | (83.7, 94.5) | (81.3, 89.7) | (46.6, 97.9) | (58.1, 69.4) | (84.9, 89.4) |
| *(Education continued from previous page)* | | | |  |  |  |  |  |  |  |  |
| Tertiary |  | 4.1* | 37.1 | 79.0 | 58.2 | 61.8 | 92.4 | 81.6 | 45.1 | 56.4 | 76.3 |
|  |  | (1.2, 7.1) | (28.3, 45.8) | (73.5, 84.6) | (12.1, 104.3) | (54.8, 68.8) | (90.0, 94.8) | (74.0, 89.3) | (29.4, 60.7) | (43.5, 69.3) | (71.1, 81.5) |
| **Employment** |  |  |  |  |  |  |  |  |  |  |  |
| Private or public employee |  | 2.9 | 49.3 | 82.7 | 69.5 | 76.9 | 93.3 | 83.6 | 72.0 | 60.5 | 84.8 |
|  |  | (-1.0, 6.7) | (40.9, 57.7) | (77.9, 87.5) | (47.5, 91.6) | (73.1, 80.7) | (88.7, 97.8) | (78.2, 89.0) | (38.4, 105.6) | (50.4, 70.5) | (80.5, 89.0) |
| Self-employed |  | 5.1 | 53.2 | 88.9 | 72.5 | 87.3 | 88.9 | 94.0 | 83.4 | 67.7 | 92.0 |
|  |  | (-0.7, 11.0) | (35.4, 71.0) | (85.3, 92.6) | (57.0, 88.0) | (85.3, 89.4) | (82.7, 95.1) | (92.1.0, 95.9) | (63.0, 103.8) | (59.2, 76.2) | (90.4, 93.5) |
| Non-income worker |  | 2.4 | 47.7 | 80.0 | 65.0 | 79.5 | 88.7 | 91.3 | 69.7 | 66.7 | 87.5 |
|  |  | (0.4, 4.3) | (34.8, 60.6) | (71.5, 88.5) | (43.2, 86.9) | (74.7, 84.3) | (83.9, 93.5) | (88.4, 94.2) | (42.4, 97.0) | (56.1, 77.2) | (84.7, 90.4) |
| No occupation |  | 4.4 | 32.1 | 81.3 | 64.1 | 62.6 | 86.5 | 69.0 | 63.9 | 50.0 | 81.1 |
|  |  | (0.9, 7.9) | (5.9, 58.4) | (73.5, 89.1) | (39.6, 88.7) | (58.6, 66.5) | (79.5, 93.4) | (57.4, 80.6) | (45.5, 82.3) | (40.1, 59.8) | (75.3, 86.9) |
| **BMI** |  |  |  |  |  |  |  |  |  |  |  |
| Underweight |  | 5.0 | 59.8 | 80.6 | 61.2 | 80.0 | 82.8 | 90.5 | 77.5 | 68.0 | 88.6 |
|  |  | (1.6, 8.4) | (47.6, 71.9) | (73.4, 87.7) | (44.8, 77.5) | (70.7, 89.3) | (64.3, 101.2) | (87.3, 93.7) | (49.4, 105.5) | (56.8, 79.1) | (84.7, 92.5) |
| Healthy weight |  | 4.8 | 51.5 | 86.1 | 72.9 | 79.3 | 89.1 | 93.2 | 81.1 | 66.0 | 90.4 |
|  |  | (1.5, 8.1) | (34.0, 69.0) | (79.9, 92.2) | (55.1, 90.7) | (74.3, 84.3) | (83.3, 94.9) | (91.1, 95.2) | (58.1, 104.1) | (58.2, 73.9) | (88.8, 91.9) |
| Overweight |  | 2.7 | 42.0 | 83.6 | 69.3 | 73.7 | 89.6 | 90.4 | 71.6 | 65.5 | 88.5 |
|  |  | (1.0, 4.4) | (31.7, 52.3) | (78.4, 88.8) | (54.0, 84.5) | (72.7, 74.7) | (84.8, 94.4) | (87.5, 93.4) | (52.7, 90.5) | (54.0, 77.0) | (85.5, 91.6) |
| Obese |  | 2.2* | 45.1 | 79.8 | 60.3 | 78.6 | 87.3 | 88.5 | 66.0 | 59.3 | 81.6 |
|  |  | (-0.7, 5.1) | (43.0, 47.2) | (73.9, 85.7) | (42.5, 78.1) | (75.8, 81.3) | (80.9, 93.7) | (84.1, 92.9) | (31.9, 100.2) | (45.1, 73.5) | (76.5, 86.7) |
| **Residence location***†* |  |  |  |  |  |  |  |  |  |  |  |
| Urban |  | N/A | 41.5 | 77.7 | N/A | N/A | N/A | 85.0 | 50.9* | 62.2 | 82.0 |
|  |  |  | (36.6, 46.5) | (69.5, 85.8) |  |  |  | (77.4, 92.7) | (46.6, 55.2) | (54.3, 70.2) | (78.7, 85.2) |
| Rural |  | N/A | 74.0* | 88.3 | N/A | N/A | N/A | 94.1 | 85.3 | 65.4 | 92.0 |
|  |  |  | (66.0, 82.0) | (84.3, 92.3) |  |  |  | (92.4, 95.8) | (74.2, 96.5) | (51.9, 79.0) | (90.5, 93.5) |

*† Kenya: N = 4138.*

*%: Adjusted prevalence based on mixed effects Poisson regression models with robust standard errors, adjusted for sex, age and clustering; 95%CI: 95% confidence interval; N/A: data not collected;* **: The primary sampling unit for the adjusted prevalence was not included to facilitate model convergence;* ‡: *The primary and secondary sampling unit for the adjusted prevalence was not included to facilitate model convergence and sex was not included as only male; BMI: body-mass index; Underweight (BMI: <18.5kg/m^2^); Healthy weight (BMI: 18.5–24.9kg/m^2^); Overweight (BMI: 25.0–29.9kg/m^2^); Obese (BMI:* $\geq$*30.0kg/m^2^).*

*^1^ Adjusted prevalence estimates for sex are based on mixed effects Poisson regression models with robust standard errors, adjusted for age and clustering.*

*^2^ Adjusted prevalence estimates for age group are based on mixed effects Poisson regression models with robust standard errors, adjusted for sex and clustering.*

*Adjusted prevalence estimates may not add up to 100% due to the effects of adjustment.*

**Figure S1.** Pooled adjusted prevalence of participation in occupational physical activity (N=26 022)

*Prevalence: pooled adjusted prevalence; 95%CI: 95% confidence interval; N: number of studies; I-squared: I^2^ statistic indicates proportion of variation due to heterogeneity; Underweight (BMI: <18.5kg/m^2^); Healthy weight (BMI: 18.5–24.9kg/m^2^); Overweight (BMI: 25.0–29.9kg/m^2^); Obese (BMI:* $\geq$*30.0kg/m^2^).*

*Residence location: N=20 022.*

*Pooled adjusted prevalence estimates are based on two-step individual pooled data meta-analysis of Poisson regression models adjusted for sex, age and clustering.*

*Sex: pooled adjusted prevalence estimates for sex are based on two-step individual pooled data meta-analysis of Poisson regression models adjusted for age and clustering.*

*Age group: pooled adjusted prevalence estimates for age group are based on two-step individual pooled data meta-analysis of Poisson regression models adjusted for sex and clustering.*

**Table S5.** Adjusted prevalence of participation in physical activity through active travel

|  |  | **DRC** | **Guinea** | **Kenya** | **Liberia** | **Seychelles** | **South Africa** | **Tanzania** | **Zanzibar** | **Togo** | **Uganda** |
| --- | --- | --- | --- | --- | --- | --- | --- | --- | --- | --- | --- |
|  |  | **N = 1502** | **N = 2125** | **N = 4184** | **N = 2206** | **N = 1232** | **N = 1014** | **N = 5525** | **N = 2640** | **N = 2051** | **N = 3543** |
|  |  | %  (95%CI) | %  (95%CI) | %  (95%CI) | %  (95%CI) | %  (95%CI) | %  (95%CI) | %  (95%CI) | %  (95%CI) | %  (95%CI) | %  (95%CI) |
| **Overall** |  | 79.7 | 90.1 | 84.9 | 76.6 | 72.9 | 94.2 | 89.9 | 91.9 | 89.2 | 86.0 |
|  |  | (73.3, 86.1) | (86.0, 94.3) | (81.8, 88.0) | (63.4, 89.8) | (67.1, 78.7) | (90.1, 98.3) | (88.3, 91.5) | (83.9, 99.9) | (85.0, 93.5) | (84.3, 87.7) |
| **Sex^1^** |  |  |  |  |  |  |  |  |  |  |  |
| Men |  | 82.6 | 91.9 | 85.7 | 76.5 | 70.2 | 95.7 | 92.8 | 92.3 | 88.5 | 87.5 |
|  |  | (75.7, 89.5) | (89.4, 94.4) | (82.3, 89.0) | (63.6, 89.5) | (63.4, 77.0) | (91.3, 100.0) | (91.1, 94.4) | (86.1, 98.5) | (84.9, 92.0) | (85.6, 89.3) |
| Women |  | 78.5 | 88.6 | 84.4 | 75.0 | 74.9 | 93.6 | 87.4 | 91.3 | 89.7 | 84.9 |
|  |  | (72.6, 84.3) | (82.8, 94.4) | (81.1, 87.7) | (63.4, 86.6) | (69.9, 79.8) | (89.5, 97.7) | (85.4, 89.5) | (82.3, 100.3) | (84.7, 94.7) | (82.8, 87.0) |
| **Age (years)^2^** |  |  |  |  |  |  |  |  |  |  |  |
| 18-24 |  | 79.4 | 92.4 | 85.3 | 60.8 | N/A | 94.9 | 91.9 | 94.7 | 92.4 | 89.3 |
|  |  | (70.5, 88.3) | (85.0, 99.8) | (81.0, 89.5) | (53.2, 68.5) |  | (90.4, 99.4) | (86.0, 97.8) | (86.8, 102.7) | (87.7, 97.2) | (86.9, 91.6) |
| 25-34 |  | 78.2 | 89.5 | 84.8 | 74.2 | 78.1 | 96.5 | 90.1 | 92.1 | 87.8 | 85.4 |
|  |  | (72.9, 83.5) | (85.2, 93.8) | (81.5, 88.1) | (61.0, 87.4) | (69.8, 86.5) | (92.6, 100.4) | (88.2, 92.0) | (84.1, 100.1) | (82.6, 93.0) | (83.0, 87.9) |
| 35-44 |  | 87.9 | 91.8 | 86.4 | 73.6 | 69.7* | 95.5 | 90.6 | 91.6 | 88.5 | 86.5 |
|  |  | (83.2, 92.7) | (86.8, 96.8) | (82.7, 90.1) | (59.9, 87.3) | (62.9, 76.5) | (92.9, 98.0) | (88.6, 92.6) | (82.8, 100.4) | (83.9, 93.2) | (83.8, 89.2) |
| 45-54 |  | 77.8 | 92.2 | 85.3 | 72.2 | 72.9 | 94.9 | 90.5 | 91.5 | 88.1 | 85.3 |
|  |  | (67.0, 88.7) | (91.5, 93.0) | (80.5, 90.1) | (58.1, 86.2) | (66.5, 79.3) | (88.3, 101.5) | (88.6, 92.4) | (85.9, 97.2) | (82.0, 94.2) | (82.0, 88.6) |
| 55-64 |  | 84.2 | 82.1 | 83.6 | 73.8 | 71.4 | 92.7 | 87.2 | 89.6 | 88.4 | 80.4 |
|  |  | (77.9, 90.4) | (82.1, 82.1) | (79.1, 88.0) | (56.1, 91.5) | (69.9, 72.9) | (85.7, 99.6) | (84.3. 90.1) | (83.2, 96.0) | (84.6, 92.1) | (75.6, 85.1) |
| 65+ |  | 67.8 | 0 | 78.8 | N/A | N/A | 85.4 | 66.7‡ | N/A | N/A | 80.8 |
|  |  | (49.7, 85.9) | (0.0, 0.0) | (71.9, 85.8) |  |  | (79.3, 91.6) | (1.3, 132.0) |  |  | (73.1, 88.5) |
| **Education** |  |  |  |  |  |  |  |  |  |  |  |
| No primary |  | 72.2 | 89.3 | 83.4 | 76.3 | N/A | 90.9 | 88.7 | 93.2 | 90.1 | 83.8 |
|  |  | (65.2, 79.1) | (79.2, 99.3) | (79.8, 86.9) | (64.0, 88.6) |  | (74.1, 107.7) | (86.6, 90.8) | (86.7, 99.7) | (85.1, 95.1) | (80.3, 87.3) |
| Primary |  | 81.2 | 88.7 | 86.8 | 75.2 | 75.6 | 90.6 | 91.2 | 91.2 | 90.3 | 87.4 |
|  |  | (75.4, 87.0) | (88.7, 88.8) | (82.7, 90.8) | (65.8, 84.7) | (72.0, 79.1) | (85.4, 95.8) | (89.6, 92.8) | (86.4, 96.1) | (86.3, 94.3) | (85.4, 89.4) |
| Secondary |  | 83.4 | 92.1 | 85.1 | 71.5 | 73.7 | 95.0 | 87.0 | 89.7 | 89.1 | 84.3 |
|  |  | (76.0, 90.9) | (90.5, 93.7) | (81.5, 88.6) | (57.9, 85.1) | (67.4, 80.0) | (91.1, 99.0) | (82.6, 91.3) | (82.9, 96.5) | (86.7, 91.6) | (81.8, 86.8) |
| *(Education continued from previous page)* | | | |  |  |  |  |  |  |  |  |
| Tertiary |  | 81.2 | 91.4 | 85.1 | 55.3 | 55.9 | 97.0 | 83.5 | 81.7 | 74.5 | 89.3 |
|  |  | (72.6, 89.7) | (90.3, 92.5) | (79.9, 90.3) | (49.1, 61.5) | (53.7, 58.1) | (93.8, 100.2) | (77.8, 89.3) | (74.1, 89.3) | (62.1, 86.9) | (85.8, 92.9) |
| **Employment** |  |  |  |  |  |  |  |  |  |  |  |
| Private or public employee |  | 84.5 | 90.7 | 85.7 | 69.5 | 77.1 | 98.1 | 87.4 | 88.0 | 80.4 | 86.2 |
|  |  | (77.5, 91.5) | (85.9, 95.5) | (81.1, 90.3) | (49.7, 89.4) | (71.2, 83.1) | (95.7, 100.5) | (83.7, 91.1) | (78.7, 97.4) | (73.0, 87.9) | (82.1, 90.3) |
| Self-employed |  | 83.3 | 92.1 | 83.8 | 77.4 | 63.9 | 98.8 | 91.4 | 93.1 | 88.7 | 85.6 |
|  |  | (76.0, 90.7) | (88.2, 96.0) | (80.3, 87.3) | (65.3, 89.5) | (53.2, 74.6) | (96.9, 100.6) | (90.0, 92.8) | (87.2, 99.1) | (85.4, 92.0) | (83.5, 87.6) |
| Non-income worker |  | 80.2 | 90.1 | 85.8 | 75.5 | 59.0 | 94.7 | 86.7 | 91.3 | 93.5 | 88.7 |
|  |  | (74.4, 85.9) | (85.6, 94.7) | (81.6, 90.0) | (59.5, 91.5) | (57.5, 60.5) | (86.7, 102.7) | (83.7, 89.8) | (83.8, 98.7) | (89.7, 97.2) | (86.3, 91.1) |
| No occupation |  | 69.7 | 72.9 | 85.3 | 64.8 | 64.0 | 92.1 | 79.6 | 88.0 | 77.8 | 76.5 |
|  |  | (63.1, 76.3) | (68.1, 77.8) | (79.8, 90.7) | (47.7, 81.8) | (59.5, 68.5) | (87.4, 96.7) | (73.2, 86.0) | (82.4, 93.5) | (71.2, 84.4) | (69.8, 83.2) |
| **BMI** |  |  |  |  |  |  |  |  |  |  |  |
| Underweight |  | 81.4 | 91.4 | 87.3 | 74.8 | 68.0 | 86.2 | 91.4 | 96.7 | 87.6 | 85.4 |
|  |  | (77.7, 85.1) | (82.0, 100.8) | (82.7, 92.0) | (69.9, 79.6) | (56.5, 79.5) | (67.2, 105.3) | (88.8, 94.0) | (94.2, 99.2) | (80.1, 95.1) | (81.2, 89.7) |
| Healthy weight |  | 81.1 | 91.1 | 85.9 | 77.4 | 77.2 | 95.0 | 91.4 | 93.6 | 91.7 | 87.6 |
|  |  | (75.0, 87.1) | (89.3, 92.9) | (82.9, 88.9) | (65.0, 89.9) | (67.0, 87.3) | (90.8, 99.2) | (89.8, 93.1) | (89.1, 98.1) | (87.3, 96.1) | (86.0, 89.3) |
| Overweight |  | 77.8 | 87.1 | 83.7 | 73.1 | 67.5 | 94.6 | 86.7 | 88.9 | 84.9 | 83.7 |
|  |  | (70.6, 85.1) | (83.4, 90.8) | (79.4, 88.0) | (54.7, 91.5) | (64.6, 70.5) | (90.6, 98.5) | (84.2, 89.2) | (82.3, 95.6) | (79.0, 90.8) | (80.4, 87.0) |
| Obese |  | 73.9 | 87.3 | 79.3 | 69.3 | 75.1 | 93.8 | 82.3 | 84.9 | 82.8 | 74.9 |
|  |  | (62.1, 85.7) | (74.1, 100.4) | (74.8, 83.8) | (54.5, 84.2) | (71.3, 78.8) | (89.6, 98.0) | (78.4, 86.1) | (72.8, 96.9) | (79.2, 86.4) | (69.1, 80.7) |
| **Residence location** *†* |  |  |  |  |  |  |  |  |  |  |  |
| Urban |  | N/A | 87.8 | 80.9 | N/A | N/A | N/A | 86.6 | 84.4* | 87.5 | 84.2 |
|  |  |  | (87.3, 88.3) | (76.3, 85.4) |  |  |  | (83.0, 90.2) | (81.4, 87.3) | (85.4, 89.6) | (80.8, 87.7) |
| Rural |  | N/A | 94.8* | 88.3 | N/A | N/A | N/A | 90.9 | 94.9 | 90.4 | 86.6 |
|  |  |  | (92.7, 97.0) | (85.7, 90.9) |  |  |  | (89.3, 92.6) | (91.6, 98.3) | (84.5, 96.3) | (84.7, 88.5) |

*† Kenya: N = 4138.*

*%: Adjusted prevalence based on mixed effects Poisson regression models with robust standard errors, adjusted for sex, age and clustering; 95%CI: 95% confidence interval; N/A: data not collected;* **: The primary sampling unit for the adjusted prevalence was not included to facilitate model convergence;* ‡: *The primary and secondary sampling unit for the adjusted prevalence was not included to facilitate model convergence and sex was not included as only male; BMI: body-mass index; Underweight (BMI: <18.5kg/m^2^); Healthy weight (BMI: 18.5–24.9kg/m^2^); Overweight (BMI: 25.0–29.9kg/m^2^); Obese (BMI:* $\geq$*30.0kg/m^2^).*

*^1^ Adjusted prevalence estimates for sex are based on mixed effects Poisson regression models with robust standard errors, adjusted for age and clustering.*

*^2^ Adjusted prevalence estimates for age group are based on mixed effects Poisson regression models with robust standard errors, adjusted for sex and clustering.*

*Adjusted prevalence estimates may not add up to 100% due to the effects of adjustment.*

**Figure S2.** Pooled adjusted prevalence of participation in active travel (N=26 022)

*Prevalence: pooled adjusted prevalence; 95%CI: 95% confidence interval; N: number of studies; I-squared: I^2^ statistic indicates proportion of variation due to heterogeneity; Underweight (BMI: <18.5kg/m^2^); Healthy weight (BMI: 18.5–24.9kg/m^2^); Overweight (BMI: 25.0–29.9kg/m^2^); Obese (BMI:* $\geq$*30.0kg/m^2^).*

*Residence location: N=20 022.*

*Pooled adjusted prevalence estimates are based on two-step individual pooled data meta-analysis of Poisson regression models adjusted for sex, age and clustering.*

*Sex: pooled adjusted prevalence estimates for sex are based on two-step individual pooled data meta-analysis of Poisson regression models adjusted for age and clustering.*

*Age group: pooled adjusted prevalence estimates for age group are based on two-step individual pooled data meta-analysis of Poisson regression models adjusted for sex and clustering.*

**Table S6.** Sensitivity analyses of mixed effects Poisson regression model for participation in leisure-time physical activity

| **Study** |  | **Sex** |  | **Age** |  | **Education** |  | **Employment** | | |  | **BMI** |  | **Residence location** |
| --- | --- | --- | --- | --- | --- | --- | --- | --- | --- | --- | --- | --- | --- | --- |
|  |  | Women |  |  |  |  |  | Self-employed | Non-income | No occupation |  |  |  | Rural |
|  |  | RR (95%CI) |  | RR (95%CI) |  | RR (95%CI) |  | RR (95%CI) | RR (95%CI) | RR (95%CI) |  | RR (95%CI) |  | RR (95%CI) |
| **Combined** |  | 0.43 (0.32, 0.60) |  | 0.80 (0.73, 0.88) |  | 1.30 (1.09, 1.55) |  | 0.83 (0.75, 0.92) | 0.99 (0.89, 1.11) | 0.85 (0.76, 0.95) |  | 1.01 (0.96, 1.08) |  | 0.88 (0.80, 0.96) |
| **Study omitted** |  |  |  |  |  |  |  |  |  |  |  |  |  |  |
| DRC |  | 0.43 (0.31, 0.60) |  | 0.79 (0.71, 0.87) |  | 1.32 (1.10, 1.59) |  | 0.85 (0.79, 0.92) | 0.99 (0.88, 1.12) | 0.85 (0.76, 0.96) |  | 1.02 (0.96, 1.08) |  | N/A |
| Guinea |  | 0.51 (0.43, 0.60) |  | 0.82 (0.77, 0.87) |  | 1.29 (1.07, 1.56) |  | 0.83 (0.73, 0.94) | 1.00 (0.89, 1.14) | 0.86 (0.73, 1.00) |  | 1.01 (0.95, 1.08) |  | 0.87 (0.79, 0.96) |
| Kenya |  | 0.42 (0.30, 0.60) |  | 0.81, 0.72, 0.90) |  | 1.29 (1.06, 1.57) |  | 0.82 (0.73, 0.92) | 0.96 (0.86, 1.08) | 0.80 (0.78, 0.81) |  | 1.02 (0.95, 1.09) |  | 0.87 (0.78, 0.96) |
| Liberia |  | 0.43 (0.31, 0.61) |  | 0.81 (0.73, 0.90) |  | 1.29 (1.06, 1.58) |  | 0.83 (0.74, 0.93) | 0.98 (0.85, 1.14) | 0.83 (0.74, 0.94) |  | 1.04 (0.99, 1.09) |  | N/A |
| Seychelles |  | 0.41 (0.29, 0.57) |  | 0.78 (0.72, 0.86) |  | 1.25 (1.18, 1.32) |  | 0.83 (0.74, 0.93) | 1.00 (0.88, 1.14) | 0.85 (0.75, 0.96) |  | 1.03 (0.96, 1.09) |  | N/A |
| South Africa |  | 0.45 (0.32, 0.63) |  | 0.80 (0.72, 0.88) |  | 1.33 (1.11, 1.59) |  | 0.82 (0.74, 0.91) | 0.98 (0.86, 1.11) | 0.86 (0.75, 0.99) |  | 1.01 (0.95, 1.09) |  | N/A |
| Tanzania |  | 0.41 (0.28, 0.60) |  | 0.80 (0.72, 0.89) |  | 1.32 (1.10, 1.58) |  | 0.83 (0.73, 0.93) | 1.03 (0.93, 1.15) | 0.85 (0.75, 0.96) |  | 1.01 (0.94, 1.08) |  | 0.85 (0.78, 0.93) |
| Zanzibar |  | 0.46 (0.33, 0.64) |  | 0.81 (0.72, 0.90) |  | 1.31 (1.09, 1.58) |  | 0.85 (0.77, 0.95) | 1.02 (0.92, 1.14) | 0.84 (0.75, 0.94) |  | 1.00 (0.95, 1.05) |  | 0.90 (0.81, 1.00) |
| Togo |  | 0.43 (0.30, 0.62) |  | 0.80 (0.72, 0.89) |  | 1.30 (1.08, 1.57) |  | 0.83 (0.74, 0.92) | 0.96 (0.86, 1.09) | 0.84 (0.74, 0.95) |  | 1.02 (0.95, 1.08) |  | 0.90 (0.82, 0.99) |
| Uganda |  | 0.42 (0.29, 0.60) |  | 0.81 (0.72, 0.90) |  | 1.30 (1.07, 1.58) |  | 0.82 (0.73, 0.91) | 0.99 (0.87, 1.13) | 0.87 (0.77, 0.97) |  | 1.00 (0.94, 1.07) |  | 0.87 (0.78, 0.97) |

*RR: Risk ratio; 95%CI: 95% confidence interval: N/A: no data on urban/rural residence collected for these studies.*

*For sex, men were the reference category; For employment, private or public employment was the reference category; For residence type, urban was the reference category.*

**Table S7.** Sensitivity analyses of mixed effects Poisson regression model for participation in occupational physical activity

| **Study** |  | **Sex** |  | **Age** |  | **Education** |  | **Employment** | | |  | **BMI** |  | **Residence location** |
| --- | --- | --- | --- | --- | --- | --- | --- | --- | --- | --- | --- | --- | --- | --- |
|  |  | Women |  |  |  |  |  | Self-employed | Non-income | No occupation |  |  |  | Rural |
|  |  | RR (95%CI) |  | RR (95%CI) |  | RR (95%CI) |  | RR (95%CI) | RR (95%CI) | RR (95%CI) |  | RR (95%CI) |  | RR (95%CI) |
| **Combined** |  | 0.97 (0.93, 1.01) |  | 0.98 (0.96, 1.00) |  | 0.96 (0.94, 0.98) |  | 1.06 (1.02, 1.11) | 0.99 (0.97, 1.02) | 0.89 (0.85, 0.94) |  | 0.97 (0.96, 0.99) |  | 1.23 (1.09, 1.39) |
| **Study omitted** |  |  |  |  |  |  |  |  |  |  |  |  |  |  |
| DRC |  | 0.97 (0.94, 1.01) |  | 0.98 (0.97, 1.00) |  | 0.96 (0.94, 0.98) |  | 1.06 (1.02, 1.11) | 0.99 (0.97, 1.02) | 0.89 (0.85, 0.94) |  | 0.98 (0.96, 0.99) |  | N/A |
| Guinea |  | 1.00 (0.97, 1.03) |  | 0.99 (0.97, 1.01) |  | 0.97 (0.94, 0.99) |  | 1.07 (1.02, 1.12) | 1.00 (0.97, 1.02) | 0.90 (0.85, 0.94) |  | 0.98 (0.96, 0.99) |  | 1.16 (1.05, 1.29) |
| Kenya |  | 0.96 (0.92, 1.01) |  | 0.98 (0.96, 1.00) |  | 0.96 (0.93, 0.98) |  | 1.07 (1.01, 1.13) | 1.00 (0.97, 1.03) | 0.88 (0.83, 0.94) |  | 0.97 (0.96, 0.99) |  | 1.27 (1.07, 1.50) |
| Liberia |  | 0.97 (0.93, 1.01) |  | 0.98 (0.96, 1.00) |  | 0.96 (0.93, 0.98) |  | 1.06 (1.02, 1.11) | 1.00 (0.97, 1.02) | 0.89 (0.85, 0.95) |  | 0.98 (0.97, 0.99) |  | N/A |
| Seychelles |  | 0.95 (0.91, 1.00) |  | 0.98 (0.96, 0.99) |  | 0.96 (0.94, 0.99) |  | 1.05 (1.01, 1.08) | 1.00 (0.96, 1.03) | 0.93 (0.90, 0.96) |  | 0.97 (0.96, 0.99) |  | N/A |
| South Africa |  | 0.97 (0.93, 1.01) |  | 0.98 (0.96, 1.00) |  | 0.95 (0.93, 0.98) |  | 1.08 (1.04, 1.12) | 1.00 (0.98, 1.03) | 0.88 (0.83, 0.94) |  | 0.97 (0.96, 0.99) |  | N/A |
| Tanzania |  | 0.95 (0.90, 1.01) |  | 0.98 (0.96, 1.00) |  | 0.95 (0.93, 0.98) |  | 1.06 (1.01, 1.11) | 0.99 (0.96, 1.01) | 0.90 (0.85, 0.95) |  | 0.97 (0.96, 0.99) |  | 1.27 (1.10, 1.47) |
| Zanzibar |  | 0.97 (0.93, 1.02) |  | 0.99 (0.97, 1.00) |  | 0.96 (0.94, 0.99) |  | 1.06 (1.01, 1.11) | 1.00 (0.97, 1.03) | 0.88 (0.83, 0.94) |  | 0.98 (0.96, 0.99) |  | 1.17 (1.06, 1.29) |
| Togo |  | 0.96 (0.92, 1.01) |  | 0.98 (0.96, 1.00) |  | 0.96 (0.93, 0.98) |  | 1.06 (1.02, 1.11) | 0.99 (0.96, 1.02) | 0.90 (0.85, 0.95) |  | 0.98 (0.96, 0.99) |  | 1.26 (1.11, 1.44) |
| Uganda |  | 0.97 (0.92, 1.01) |  | 0.98 (0.96, 1.00) |  | 0.96 (0.93, 0.99) |  | 1.07 (1.02, 1.13) | 1.00 (0.96, 1.03) | 0.89 (0.84, 0.94) |  | 0.97 (0.96, 0.99) |  | 1.27 (1.08, 1.50) |

*RR: Risk ratio; 95%CI: 95% confidence interval: N/A: no data on urban/rural residence collected for these studies.*

*For sex, men were the reference category; For employment, private or public employment was the reference category; For residence type, urban was the reference category.*

**Table S8.** Sensitivity analyses of mixed effects Poisson regression model for participation in active travel

| **Study** |  | **Sex** |  | **Age** |  | **Education** |  | **Employment** | | |  | **BMI** |  | **Residence location** |
| --- | --- | --- | --- | --- | --- | --- | --- | --- | --- | --- | --- | --- | --- | --- |
|  |  | Women |  |  |  |  |  | Self-employed | Non-income | No occupation |  |  |  | Rural |
|  |  | RR (95%CI) |  | RR (95%CI) |  | RR (95%CI) |  | RR (95%CI) | RR (95%CI) | RR (95%CI) |  | RR (95%CI) |  | RR (95%CI) |
| **Combined** |  | 0.98 (0.96, 1.01) |  | 0.99 (0.99, 1.00) |  | 0.99 (0.97, 1.01) |  | 1.02 (0.99, 1.05) | 0.99 (0.94, 1.05) | 0.91 (0.87, 0.96) |  | 0.97 (0.96, 0.99) |  | 1.06 (1.03, 1.09) |
| **Study omitted** |  |  |  |  |  |  |  |  |  |  |  |  |  |  |
| DRC |  | 0.98 (0.96, 1.01) |  | 0.99 (0.99, 1.00) |  | 0.98 (0.97, 1.00) |  | 1.02 (0.99, 1.05) | 1.00 (0.94, 1.06) | 0.92 (0.87, 0.97) |  | 0.98 (0.96, 0.99) |  | N/A |
| Guinea |  | 0.98 (0.96, 1.01) |  | 0.99 (0.99, 1.00) |  | 0.99 (0.97, 1.01) |  | 1.02 (0.99, 1.05) | 0.99 (0.93, 1.06) | 0.92 (0.87, 0.96) |  | 0.97 (0.96, 0.99) |  | 1.05 (1.01, 1.09) |
| Kenya |  | 0.98 (0.95, 1.01) |  | 0.99 (0.99, 1.00) |  | 0.99 (0.97, 1.01) |  | 1.02 (0.99, 1.05) | 0.99 (0.94, 1.05) | 0.90 (0.86, 0.95) |  | 0.98 (0.96 (0.99) |  | 1.05 (1.00, 1.09) |
| Liberia |  | 0.99 (0.96, 1.01) |  | 0.99 (0.99, 1.00) |  | 0.99 (0.97, 1.01) |  | 1.01 (0.99, 1.04) | 0.98 (0.93, 1.04) | 0.91 (0.86, 0.96) |  | 0.98 (0.96, 0.99) |  | N/A |
| Seychelles |  | 0.97 (0.96, 0.99) |  | 0.99 (0.99, 1.00) |  | 1.00 (0.99, 1.01) |  | 1.03 (1.01, 1.05) | 1.02 (0.99, 1.05) | 0.93 (0.89, 0.97) |  | 0.97 (0.96, 0.99) |  | N/A |
| South Africa |  | 0.98 (0.96, 1.01) |  | 0.99 (0.99, 1.00) |  | 0.99 (0.97, 1.01) |  | 1.02 (0.98, 1.06) | 1.00 (0.94, 1.06) | 0.90 (0.85, 0.96) |  | 0.97 (0.96, 0.98) |  | N/A |
| Tanzania |  | 0.99 (0.96, 1.01) |  | 0.99 (0.99, 1.00) |  | 0.99 (0.97, 1.01) |  | 1.02 (0.99, 1.05) | 0.99 (0.93, 1.06) | 0.91 (0.86, 0.96) |  | 0.98 (0.96, 0.99) |  | 1.07 (1.03, 1.10) |
| Zanzibar |  | 0.98 (0.95, 1.01) |  | 0.99 (0.99,1.00) |  | 0.99 (0.96, 1.02) |  | 1.02 (0.99, 1.05) | 0.99 (0.93, 1.06) | 0.90 (0.86, 0.95) |  | 0.98 (0.96, 0.99) |  | 1.05 (1.01, 1.09) |
| Togo |  | 0.98 (0.96, 1.01) |  | 0.99 (0.99, 0.99) |  | 0.99 (0.97, 1.01) |  | 1.01 (0.99, 1.04) | 0.98 (0.93, 1.04) | 0.91 (0.86, 0.96) |  | 0.98 (0.96, 0.99) |  | 1.07 (1.04, 1.10) |
| Uganda |  | 0.98 (0.96, 1.01) |  | 0.99 (0.99, 1.00) |  | 0.99 (0.97, 1.01) |  | 1.02 (0.99, 1.05) | 0.99 (0.93, 1.05) | 0.91 (0.86, 0.96) |  | 0.98 (0.97, 0.99) |  | 1.07 (1.03, 1.10) |

*RR: Risk ratio; 95%CI: 95% confidence interval: N/A: no data on urban/rural residence collected for these studies.*

*For sex, men were the reference category; For employment, private or public employment was the reference category; For residence type, urban was the reference category.*

**Figure S3**. Association between sex and participation in leisure-time physical activity, adjusted for physical activity at work and through travel (N=26 022)

*Men: N = 10 878; Women: N = 15 144.*

*RR: Risk ratio; 95%CI: 95% confidence interval; N: number of studies in two-step individual participant data (IPD) meta-analysis; P: corresponds to the Z-test of significance for the pooled RR; I-squared: I^2^ statistic indicates proportion of variation due to heterogeneity; P for heterogeneity: corresponds to the P value for the chi-squared test of heterogeneity.*

*Two-step IPD meta-analysis of RRs calculated by DerSimonian and Laird random effects method. Individual study RRs calculated by multivariable mixed effects Poisson regression models with robust standard errors.*

*Categorical variables for age, education and body-mass index (BMI) were included in the model as continuous variables.*

*Men: were the reference for women.*

*Multivariate recreation model: RRs calculated by multivariable mixed effects Poisson regression models adjusted for age, education, employment and BMI.*

*Adjusted for work (binary): RRs calculated by multivariable mixed effects Poisson regression models adjusted for age, education, employment, BMI and participation in occupational physical activity.*

*Adjusted for work (continuous): RRs calculated by multivariable mixed effects Poisson regression models adjusted for age, education, employment, BMI and MET-minutes spent in occupational physical activity.*

*Adjusted for travel (binary): RRs calculated by multivariable mixed effects Poisson regression models adjusted for age, education, employment, BMI and participation in active travel.*

*Adjusted for travel (continuous): RRs calculated by multivariable mixed effects Poisson regression models adjusted for age, education, employment, BMI and MET-minutes spent in active travel.*

*Adjusted for work & travel (binary): RRs calculated by multivariable mixed effects Poisson regression models adjusted for age, education, employment, BMI, participation in occupational physical activity and participation in active travel.*

*Adjusted for work & travel (continuous): RRs calculated by multivariable mixed effects Poisson regression models adjusted for age, education, employment, BMI, MET-minutes spent in occupational physical activity and MET-minutes spent in active travel.*

**Figure S4**. Association between age and participation in leisure-time physical activity, adjusted for physical activity at work and through travel (N=26 022)

*RR: Risk ratio; 95%CI: 95% confidence interval; N: number of studies in two-step IPD meta-analysis; P: corresponds to the Z-test of significance for the pooled RR; I-squared: I^2^ statistic indicates proportion of variation due to heterogeneity; P for heterogeneity: corresponds to the P value for the chi-squared test of heterogeneity.*

*Two-step IPD meta-analysis of RRs calculated by DerSimonian and Laird random effects method. Individual study RRs calculated by multivariable mixed effects Poisson regression models with robust standard errors.*

*Categorical variables for age, education and body-mass index (BMI) were included in the model as continuous variables.*

*Multivariate recreation model: RRs calculated by multivariable mixed effects Poisson regression models adjusted for sex, education, employment and BMI.*

*Adjusted for work (binary): RRs calculated by multivariable mixed effects Poisson regression models adjusted for sex, education, employment, BMI and participation in occupational physical activity.*

*Adjusted for work (continuous): RRs calculated by multivariable mixed effects Poisson regression models adjusted for sex, education, employment, BMI and MET-minutes spent in occupational physical activity.*

*Adjusted for travel (binary): RRs calculated by multivariable mixed effects Poisson regression models adjusted for sex, education, employment, BMI and participation in active travel.*

*Adjusted for travel (continuous): RRs calculated by multivariable mixed effects Poisson regression models adjusted for sex, education, employment, BMI and MET-minutes spent in active travel.*

*Adjusted for work & travel (binary): RRs calculated by multivariable mixed effects Poisson regression models adjusted for sex, education, employment, BMI, participation in occupational physical activity and participation in active travel.*

*Adjusted for work & travel (continuous): RRs calculated by multivariable mixed effects Poisson regression models adjusted for sex, education, employment, BMI, MET-minutes spent in occupational physical activity and MET-minutes spent in active travel.*

**Figure S5**. Association between education and participation in leisure-time physical activity, adjusted for physical activity at work and through travel (N=26 022)

*RR: Risk ratio; 95%CI: 95% confidence interval; N: number of studies in two-step IPD meta-analysis; P: corresponds to the Z-test of significance for the pooled RR; I-squared: I^2^ statistic indicates proportion of variation due to heterogeneity; P for heterogeneity: corresponds to the P value for the chi-squared test of heterogeneity.*

*Two-step IPD meta-analysis of RRs calculated by DerSimonian and Laird random effects method. Individual study RRs calculated by multivariable mixed effects Poisson regression models with robust standard errors.*

*Categorical variables for age, education and body-mass index (BMI) were included in the model as continuous variables.*

*Multivariate recreation model: RRs calculated by multivariable mixed effects Poisson regression models adjusted for sex, age, employment and BMI.*

*Adjusted for work (binary): RRs calculated by multivariable mixed effects Poisson regression models adjusted for sex, age, employment, BMI and participation in occupational physical activity.*

*Adjusted for work (continuous): RRs calculated by multivariable mixed effects Poisson regression models adjusted for sex, age, employment, BMI and MET-minutes spent in occupational physical activity.*

*Adjusted for travel (binary): RRs calculated by multivariable mixed effects Poisson regression models adjusted for sex, age, employment, BMI and participation in active travel.*

*Adjusted for travel (continuous): RRs calculated by multivariable mixed effects Poisson regression models adjusted for sex, age, employment, BMI and MET-minutes spent in active travel.*

*Adjusted for work & travel (binary): RRs calculated by multivariable mixed effects Poisson regression models adjusted for sex, age, employment, BMI, participation in occupational physical activity and participation in active travel.*

*Adjusted for work & travel (continuous): RRs calculated by multivariable mixed effects Poisson regression models adjusted for sex, age, employment, BMI, MET-minutes spent in occupational physical activity and MET-minutes spent in active travel.*

**Figure S6**. Association between body-mass index and participation in leisure-time physical activity, adjusted for physical activity at work and through travel (N=26 022)

*RR: Risk ratio; 95%CI: 95% confidence interval; N: number of studies in two-step IPD meta-analysis; P: corresponds to the Z-test of significance for the pooled RR; I-squared: I^2^ statistic indicates proportion of variation due to heterogeneity; P for heterogeneity: corresponds to the P value for the chi-squared test of heterogeneity.*

*Two-step IPD meta-analysis of RRs calculated by DerSimonian and Laird random effects method. Individual study RRs calculated by multivariable mixed effects Poisson regression models with robust standard errors.*

*Categorical variables for age, education and body-mass index (BMI) were included in the model as continuous variables.*

*Multivariate recreation model: RRs calculated by multivariable mixed effects Poisson regression models adjusted for sex, age, education and employment.*

*Adjusted for work (binary): RRs calculated by multivariable mixed effects Poisson regression models adjusted for sex, age, education, employment and participation in occupational physical activity.*

*Adjusted for work (continuous): RRs calculated by multivariable mixed effects Poisson regression models adjusted for sex, age, education, employment and MET-minutes spent in occupational physical activity.*

*Adjusted for travel (binary): RRs calculated by multivariable mixed effects Poisson regression models adjusted for sex, age, education, employment and participation in active travel.*

*Adjusted for travel (continuous): RRs calculated by multivariable mixed effects Poisson regression models adjusted for sex, age, education, employment and MET-minutes spent in active travel.*

*Adjusted for work & travel (binary): RRs calculated by multivariable mixed effects Poisson regression models adjusted for sex, age, education, employment, participation in occupational physical activity and participation in active travel.*

*Adjusted for work & travel (continuous): RRs calculated by multivariable mixed effects Poisson regression models adjusted for sex, age, education, employment, MET-minutes spent in occupational physical activity and MET-minutes spent in active travel.*

**Figure S7**. Association between employment categories and participation in leisure-time physical activity, adjusted for physical activity at work and through travel (N=26 022)

*RR: Risk ratio; 95%CI: 95% confidence interval; N: number of studies in two-step IPD meta-analysis; P: corresponds to the Z-test of significance for the pooled RR; I-squared: I^2^ statistic indicates proportion of variation due to heterogeneity; P for heterogeneity: corresponds to the P value for the chi-squared test of heterogeneity.*

*Two-step IPD meta-analysis of RRs calculated by DerSimonian and Laird random effects method. Individual study RRs calculated by multivariable mixed effects Poisson regression models with robust standard errors.*

*Categorical variables for age, education and body-mass index (BMI) were included in the model as continuous variables.*

*Public and private employee were the reference for all employment categories.*

*Multivariate recreation model: RRs calculated by multivariable mixed effects Poisson regression models adjusted for sex, age, education and BMI.*

*Adjusted for work (binary): RRs calculated by multivariable mixed effects Poisson regression models adjusted for sex, age, education, BMI and participation in occupational physical activity.*

*Adjusted for work (continuous): RRs calculated by multivariable mixed effects Poisson regression models adjusted for sex, age, education, BMI and MET-minutes spent in occupational physical activity.*

*Adjusted for travel (binary): RRs calculated by multivariable mixed effects Poisson regression models adjusted for sex, age, education, BMI and participation in active travel.*

*Adjusted for travel (continuous): RRs calculated by multivariable mixed effects Poisson regression models adjusted for sex, age, education, BMI and MET-minutes spent in active travel.*

*Adjusted for work & travel (binary): RRs calculated by multivariable mixed effects Poisson regression models adjusted for sex, age, education, BMI, participation in occupational physical activity and participation in active travel.*

*Adjusted for work & travel (continuous): RRs calculated by multivariable mixed effects Poisson regression models adjusted for sex, age, education, BMI, MET-minutes spent in occupational physical activity and MET-minutes spent in active travel.*

**Figure S8**. Association between residence location and participation in leisure-time physical activity, adjusted for physical activity at work and through travel (N=20 022)

*RR: Risk ratio; 95%CI: 95% confidence interval; N: number of studies in two-step IPD meta-analysis; P: corresponds to the Z-test of significance for the pooled RR; I-squared: I^2^ statistic indicates proportion of variation due to heterogeneity; P for heterogeneity: corresponds to the P value for the chi-squared test of heterogeneity.*

*Two-step IPD meta-analysis of RRs calculated by DerSimonian and Laird random effects method. Individual study RRs calculated by multivariable mixed effects Poisson regression models with robust standard errors.*

*Categorical variables for age, education and body-mass index (BMI) were included in the model as continuous variables.*

*Urban residents: were the reference for rural residents.*

*Multivariate recreation model: RRs calculated by multivariable mixed effects Poisson regression models adjusted for sex, age, education, employment and BMI.*

*Adjusted for work (binary): RRs calculated by multivariable mixed effects Poisson regression models adjusted for sex, age, education, employment, BMI and participation in occupational physical activity.*

*Adjusted for work (continuous): RRs calculated by multivariable mixed effects Poisson regression models adjusted for sex, age, education, employment, BMI and MET-minutes spent in occupational physical activity.*

*Adjusted for travel (binary): RRs calculated by multivariable mixed effects Poisson regression models adjusted for sex, age, education, employment, BMI and participation in active travel.*

*Adjusted for travel (continuous): RRs calculated by multivariable mixed effects Poisson regression models adjusted for sex, age, education, employment, BMI and MET-minutes spent in active travel.*

*Adjusted for work & travel (binary): RRs calculated by multivariable mixed effects Poisson regression models adjusted for sex, age, education, employment, BMI, participation in occupational physical activity and participation in active travel.*

*Adjusted for work & travel (continuous): RRs calculated by multivariable mixed effects Poisson regression models adjusted for sex, age, education, employment, BMI, MET-minutes spent in occupational physical activity and MET-minutes spent in active travel.*

**Table S10.** Meta-regression of RRs for participation in leisure time physical activity

|  | **Sex** | | **Age** | | **Education** | | **Employment** | | | | | | **BMI** | | **Residence location** | |
| --- | --- | --- | --- | --- | --- | --- | --- | --- | --- | --- | --- | --- | --- | --- | --- | --- |
|  | Women | |  | |  | | Self-employed | | Non-income work | | No occupation | |  | | Rural | |
| No. of studies | 10 | | 10 | | 10 | | 10 | | 10 | | 10 | | 10 | | 6 | |
| Unadjusted I^2^ % | 97.6% | | 97.1% | | 98.1% | | 75.7% | | 66.7% | | 58.6% | | 79.4% | | 62.1% | |
|  | $\boldsymbol{\beta}$  **(95%CI)** | **Adj.R^2^**  **(%)** | $\boldsymbol{\beta}$ **(95%CI)** | **Adj.R^2^**  **(%)** | $\boldsymbol{\beta}$ **(95%CI)** | **Adj.R^2^**  **(%)** | $\boldsymbol{\beta}$  **(95%CI)** | **Adj.R^2^**  **(%)** | $\boldsymbol{\beta}$  **(95%CI)** | **Adj.R^2^**  **(%)** | $\boldsymbol{\beta}$ **(95%CI)** | **Adj.R^2^**  **(%)** | $\boldsymbol{\beta}$ **(95%CI)** | **Adj.R^2^**  **(%)** | $\boldsymbol{\beta}$ **(95%CI)** | **Adj.R^2^**  **(%)** |
| Income group | 1.1  (0.8, 1.7) | -5.4 | 1.1  (1.0, 1.2) | 22.2 | 1.1  (0.9, 1.3) | 9.8 | 1.1  (0.9, 1.4) | -14.3 | 1.1  (0.9, 1.3) | -16.4 | 1.0  (0.8, 1.2) | -35.3 | 1.0  (0.9, 1.1) | -4.1 | 1.1  (0.8, 1.5) | -27.7 |
| Study year | 1.1  (0.9, 1.2) | 9.8 | 1.0  (1.0, 1.0) | -15.4 | 1.0  (1.0, 1.1) | -2.1 | 1.1  (1.0, 1.1)* | 24.8 | 1.0  (1.0, 1.1) | -10.2 | 1.0  (1.0, 1.1) | -0.8 | 1.0  (1.0, 1.0) | -14.6 | 1.0  (1.0, 1.1) | 51.7 |
| Study size | 1.0  (1.0, 2.0) | -3.3 | 1.0  (1.0, 1.0) | -13.2 | 1.0  (1.0, 1.0) | -5.1 | 1.0  (1.0, 1.0) | -105.7 | 1.0  (1.0, 1.0) | -11.2 | 1.0  (1.0, 1.0) | 21.5 | 1.0  (1.0, 1.0) | -7.8 | 1.0  (1.0, 1.0) | 100.0 |
| Study scope | 0.5  (0.3, 0.8) | 66.9 | 1.0  (0.8, 1.2) | -9.1 | 0.8  (0.7, 1.1) | 11.2 | 0.9  (0.7, 1.1) | 68.1 | 0.9  (0.6, 1.3) | -13.6 | 0.9  (0.7, 1.2) | 22.0 | 1.1  (0.9, 1.2) | -17.9 | 1.0  (0.7, 1.3) | -21.7 |
| GDP per capita | 1.0  (1.0, 1.0) | 2.0 | 1.0  (1.0, 1.0) | 35.7 | 1.0  (1.0, 1.0) | 48.8 | 1.0  (1.0, 1.0) | -90.9 | 1.0  (1.0, 1.0) | -23.3 | 1.0  (1.0, 1.0) | -26.3 | 1.0  (1.0, 1.0) | -0.2 | 1.0  (1.0, 1.0) | 17.9 |
| HDI | 4.7  (0.4, 56.7) | 16.5 | 1.9  (1.0, 3.9) | 56.1 | 2.1  (0.8, 5.8) | 20.6 | 2.1  (0.4, 11.9) | -73.1 | 1.1  (0.3, 4.2) | -23.3 | 1.1  (0.3, 3.7) | -37.0 | 0.94  (0.5, 1.8) | -13.5 | 1.7  (0.1, 41.8) | -12.9 |
| Urban Population | 1.0  (1.0, 1.0) | -13.5 | 1.0  (1.0, 1.0) | -0.6 | 1.0  (1.0, 1.0) | -8.3 | 1.0  (1.0, 1.0) | -99.6 | 1.0  (1.0, 1.0) | -22.8 | 1.0  (1.0, 1.0) | -24.4 | 1.0  (1.0, 1.0) | 35.4 | 1.0  (1.0, 1.0) | 14.6 |

*β: meta-regression coefficient; 95%CI: Confidence Interval; Adj.R^2^: Adjusted residual R^2^ is the proportion of between-study variance explained by the covariates; Negative Adj.R^2^means the potential explanatory increased the heterogeneity; * P value<0.01 after Monte Carlo permutation analyses ** P value<0.001 after Monte Carlo permutation analyses.*

*For sex, men were the reference category; For age, 25-34 years was the reference category; For employment, private or public employment was the reference category; For residence location, urban was the reference category.*

*GDP per capita: Global domestic product per capita; HDI: Human Development Index; Urban population: Percentage of the total population living in urban areas.*

**Table S11.** Meta-regression of RRs for participation in occupational physical activity

|  | **Sex** | | **Age** | | **Education** | | **Employment** | | | | | | **BMI** | | **Residence location** | |
| --- | --- | --- | --- | --- | --- | --- | --- | --- | --- | --- | --- | --- | --- | --- | --- | --- |
|  | Women | |  | |  | | Self-employed | | Non-income work | | No occupation | |  | | Rural | |
| No. of studies | 10 | | 10 | | 10 | | 10 | | 10 | | 10 | | 10 | | 6 | |
| Unadjusted I^2^ % | 91.7% | | 96.0% | | 91.4% | | 68.1% | | 26.0% | | 54.1% | | 68.7% | | 93.3% | |
|  | $\boldsymbol{\beta}$ **(95%CI)** | **Adj.R^2^**  **(%)** | $\boldsymbol{\beta}$ **(95%CI)** | **Adj.R^2^**  **(%)** | $\boldsymbol{\beta}$ **(95%CI)** | **Adj.R^2^**  **(%)** | $\boldsymbol{\beta}$ **(95%CI)** | **Adj.R^2^**  **(%)** | $\boldsymbol{\beta}$  **(95%CI)** | **Adj.R^2^**  **(%)** | $\boldsymbol{\beta}$ **(95%CI)** | **Adj.R^2^**  **(%)** | $\boldsymbol{\beta}$ **(95%CI)** | **Adj.R^2^**  **(%)** | $\boldsymbol{\beta}$ **(95%CI)** | **Adj.R^2^**  **(%)** |
| Income group | 1.0  (0.9, 1.2) | -6.1 | 1.0  (1.0, 1.0) | 25.5 | 1.0  (1.0, 1.0) | -14.1 | 1.0  (0.9, 1.1) | -22.8 | 1.0  (0.9, 1.0) | -15.4 | 1.0  (0.9, 1.1) | -25.4 | 1.0  (1.0, 1.0) | 21.5 | 0.9  (0.4, 1.7) | -18.8 |
| Study year | 1.0  (1.0, 1.1) | 23.4 | 1.0  (1.0, 1.0) | 55.3 | 1.0  (1.0, 1.0) | -12.2 | 1.0  (1.0, 1.0) | -13.2 | 1.0  (1.0, 1.0) | 17.6 | 1.0  (1.0, 1.1) | -14.7 | 1.0  (1.0, 1.0) | 27.3 | 0.9  (0.9, 1.0) | 28.4 |
| Study size | 1.0  (1.0, 1.0) | -17.8 | 1.0  (1.0, 1.0) | -16.1 | 1.0  (1.0, 1.0) | 5.4 | 1.0  (1.0, 1.0) | -22.0 | 1.0  (1.0, 1.0) | -4.3 | 1.0  (1.0, 1.0) | -22.9 | 1.0  (1.0, 1.0) | -36.5 | 1.0  (1.0, 1.0) | 16.8 |
| Study scope | 0.8  (0.7, 1.0) | 60.8 | 1.0  (0.9, 1.0) | 66.3 | 1.0  (0.9, 1.1) | -20.3 | 0.9  (0.8, 1.0) | 42.3 | 0.9  (0.9, 1.0) | 0.5 | 1.1  (0.9, 1.2) | 10.1 | 1.0  (0.9, 1.0) | -33.3 | 1.5  (1.3, 1.6) | 100.0 |
| GDP per capita | 1.0  (1.0, 1.0) | -3.1 | 1.0  (1.0, 1.0) | 20.9 | 1.0  (1.0, 1.0) | -11.9 | 1.0  (1.0, 1.0) | -3.3 | 1.0  (1.0, 1.0) | -65.0 | 1.0  (1.0, 1.0) | 45.5 | 1.0  (1.0, 1.0) | 0.9 | 1.0  (1.0, 1.0) | -18.3 |
| HDI | 1.7  (0.9, 3.6) | 25.0 | 1.1  (1.0, 1.3) | 43.3 | 1.0  (0.8, 1.4) | -16.1 | 1.1  (0.7, 1.8) | -10.1 | 0.9  (0.7, 1.3) | -50.4 | 0.8  (0.4, 1.4) | 4.8 | 1.1  (1.0, 1.3) | 53.3 | 0.1  (0.0, 6.3) | 21.9 |
| Urban Population | 1.0  (1.0, 1.0) | -21.6 | 1.0  (1.0, 1.0) | -6.2 | 1.0  (1.0, 1.0) | -14.4 | 1.0  (1.0, 1.0) | -24.6 | 1.0  (1.0, 1.0) | -39.0 | 1.0  (1.0, 1.0) | 23.1 | 1.0  (1.0, 1.0) | -43.0 | 1.0  (1.0, 1.0) | -12.5 |

*β: meta-regression coefficient; 95%CI: Confidence Interval; Adj.R^2^: Adjusted residual R^2^ is the proportion of between-study variance explained by the covariates; Negative Adj.R^2^means the potential explanatory increased the heterogeneity; * P value<0.01 after Monte Carlo permutation analyses ** P value<0.001 after Monte Carlo permutation analyses.*

*For sex, men were the reference category; For age, 25-34 years was the reference category; For employment, private or public employment was the reference category; For residence type, urban was the reference category.*

*GDP per capita: Global domestic product per capita; HDI: Human Development Index; Urban population: Percentage of the total population living in urban areas.*

**Table S12.** Meta-regression of RRs for participation in physical activity through active travel

|  | **Sex** | | **Age** | | **Education** | | **Employment** | | | | | | **BMI** | | **Residence location** | |
| --- | --- | --- | --- | --- | --- | --- | --- | --- | --- | --- | --- | --- | --- | --- | --- | --- |
|  | Women | |  | |  | | Self-employed | | Non-income work | | No occupation | |  | | Rural | |
| No. of studies | 10 | | 10 | | 10 | | 10 | | 10 | | 10 | | 10 | | 6 | |
| Unadjusted I^2^ % | 94.9% | | 0.0% | | 90.4% | | 63.2% | | 87.5% | | 81.7% | | 37.4% | | 76.1% | |
|  | $\boldsymbol{\beta}$  **(95%CI)** | **Adj.R^2^**  **(%)** | $\boldsymbol{\beta}$ **(95%CI)** | **Adj.R^2^**  **(%)** | $\boldsymbol{\beta}$  **(95%CI)** | **Adj.R^2^**  **(%)** | $\boldsymbol{\beta}$ **(95%CI)** | **Adj.R^2^**  **(%)** | $\boldsymbol{\beta}$  **(95%CI)** | **Adj.R^2^**  **(%)** | $\boldsymbol{\beta}$ **(95%CI)** | **Adj.R^2^**  **(%)** | $\boldsymbol{\beta}$ **(95%CI)** | **Adj.R^2^**  **(%)** | $\boldsymbol{\beta}$ **(95%CI)** | **Adj.R^2^**  **(%)** |
| Income group | 1.0  (1.0, 1.0) | 38.0 | 1.0  (1.0, 1.0) | 0.0 | 1.0  (0.9, 1.0) | 11.9 | 1.0  (0.9, 1.0) | -72.1 | 0.9  (0.8, 1.0)* | 55.7 | 1.0  (0.9, 1.1) | -14.3 | 1.0  (1.0, 1.0) | 100.0 | 1.0  (0.9, 1.2) | -6.0 |
| Study year | 1.0  (1.0, 1.0) | 6.5 | 1.0  (1.0, 1.0) | 0.0 | 1.0  (1.0, 1.0) | -4.0 | 1.0  (1.0, 1.0) | 100.0 | 1.0  (1.0, 1.0) | -11.6 | 1.0  (1.0, 1.0) | 3.9 | 1.0  (1.0, 1.0) | -27.2 | 1.0  (1.0, 1.0) | -38.7 |
| Study size | 1.0  (1.0, 1.0) | 4.5 | 1.0  (1.0, 1.0) | 0.0 | 1.0  (1.0, 1.0) | -8.2 | 1.0  (1.0, 1.0) | -150.0 | 1.0  (1.0, 1.0) | -4.3 | 1.0  (1.0, 1.0) | 4.3 | 1.0  (1.0, 1.0) | 36.7 | 1.0  (1.0, 1.0) | -26.2 |
| Study scope | 1.0  (0.9, 1.0) | -8.9 | 1.0  (1.0, 1.0) | 0.0 | 1.0  (1.0, 1.1) | 4.9 | 1.0  (0.9, 1.1) | -134.2 | 1.0  (0.8, 1.2) | -14.9 | 1.0  (0.9, 1.1) | -15.9 | 1.0  (1.0, 1.0) | 100.0 | 1.1  (1.0, 1.1) | 40.0 |
| GDP per capita | 1.0  (1.0, 1.0) | 69.2 | 1.0  (1.0, 1.0) | 0.0 | 1.0  (1.0, 1.0) | 65.4 | 1.0  (1.0, 1.0) | -7.0 | 1.0  (1.0, 1.0)* | 88.5 | 1.0  (1.0, 1.0) | 15.3 | 1.0  (1.0, 1.0) | 97.8 | 1.0  (1.0, 1.0) | -9.1 |
| HDI | 1.2  (1.1, 1.4) | 60.1 | 1.0  (0.9, 1.0) | 0.0 | 0.8  (0.6, 1.0) | 41.6 | 0.7  (0.5, 1.1) | 33.3 | 0.5  (0.3, 0.8) | 55.1 | 1.0  (0.6, 1.6) | -14.8 | 1.1  (1.0, 1.2) | 42.2 | 1.0  (0.4, 2.5) | -41.6 |
| Urban Population | 1.0  (1.0, 1.0) | -4.6 | 1.0  (1.0, 1.0) | 0.0 | 1.0  (1.0, 1.0) | -2.2 | 1.0  (1.0, 1.0) | -156.7 | 1.0  (1.0, 1.0) | 4.3 | 1.0  (1.0, 1.0) | -11.4 | 1.0  (1.0, 1.0)* | 100.0 | 1.0  (1.0, 1.0) | -32.6 |

*β: meta-regression coefficient; 95%CI: Confidence Interval; Adj.R^2^: Adjusted residual R^2^ is the proportion of between-study variance explained by the covariates; Negative Adj.R^2^means the potential explanatory increased the heterogeneity; * P value<0.01 after Monte Carlo permutation analyses ** P value<0.001 after Monte Carlo permutation analyses.*

*For sex, men were the reference category; For age, 25-34 years was the reference category; For employment, private or public employment was the reference category; For residence type, urban was the reference category.*

*GDP per capita: Global domestic product per capita; HDI: Human Development Index; Urban population: Percentage of the total population living in urban areas.*

**Figure S9.** Association between sociodemographic characteristics and participation in leisure-time physical activity by education

*Primary not completed: N = 8802; Primary: N = 8607; Secondary: N = 6810; Tertiary: N = 1803.*

*RR: Pooled risk ratio; 95%CI: 95% confidence interval; N: number of studies in two-step IPD meta-analysis; P: corresponds to the Z-test of significance for the pooled RR; I-squared: I^2^ statistic indicates proportion of variation due to heterogeneity; P for heterogeneity: corresponds to the P value for the chi-squared test of heterogeneity; P for interaction: corresponds to the Z-test of significance for the pooled interaction term.*

*Pooled RRs calculated from two-step individual participant data (IPD) meta-analysis of multivariable mixed effects Poisson regression models with robust standard errors, adjusted for levels of clustering and all other covariates in the figure except residence location.*

*Categorical variables for age and BMI were included in the model as continuous variables.*

*Men were the reference for women; public and private employees were the reference for employment; urban residents were the reference for rural residents.*

*Sensitivity analyses revealed that Seychelles influenced the pooled RR for non-income work in those with a tertiary education. Excluding Seychelles, attenuated the RR for non-income work towards the null (RR: 1.11; 95%CI: 0.96, 1.28; P=0.161; I^2^=51.0%; P for heterogeneity=0.046; P for interaction=0.017). Likewise, DRC influenced the pooled RR for those with no occupation and a secondary education, once excluded the RR for this group was no longer statistically different compared with public or private employees (RR: 0.91; 95%CI: 0.82, 1.01; P=0.077; I^2^=0.0%; P for heterogeneity=0.625; P for interaction=0.100).*

**Figure S10.** Association between sociodemographic characteristics and participation in leisure-time physical activity by residence location

*Urban: N = 7311; Rural: N = 12711.*

*RR: Pooled risk ratio; 95%CI: 95% confidence interval; N: number of studies in two-step IPD meta-analysis; P: corresponds to the Z-test of significance for the pooled RR; I-squared: I^2^ statistic indicates proportion of variation due to heterogeneity; P for heterogeneity: corresponds to the P value for the chi-squared test of heterogeneity; P for interaction: corresponds to the Z-test of significance for the pooled interaction term.*

*Pooled RRs calculated from two-step individual participant data (IPD) meta-analysis of multivariable mixed effects Poisson regression models with robust standard errors, adjusted for levels of clustering and all other covariates in the figure except residence location.*

*Categorical variables for age, education and BMI were included in the model as continuous variables.*

*Men were the reference for women; public and private employees were the reference for employment.*

*Sensitivity analyses revealed no evidence of outlier studies (data not shown).*
